# Rational design of induced regeneration via somatic embryogenesis in the absence of exogenous phytohormones

**DOI:** 10.1093/plcell/koaf252

**Published:** 2025-10-19

**Authors:** Jana Wittmer, Menno Pijnenburg, Tristan Wijsman, Sieme Pelzer, Kelvin Adema, Merijn Kerstens, An-Nikol Kutevska, Joke Fierens, Hugo Hofhuis, Robert Sevenier, Bjorn Kloosterman, Michiel de Both, Wouter Kohlen, Harm Nijveen, Ben Scheres, Renze Heidstra

**Affiliations:** Cell and Developmental Biology, Cluster Plant Developmental Biology, Wageningen University & Research, Droevendaalsesteeg 1, Wageningen 6708PB, The Netherlands; Cell and Developmental Biology, Cluster Plant Developmental Biology, Wageningen University & Research, Droevendaalsesteeg 1, Wageningen 6708PB, The Netherlands; Cell and Developmental Biology, Cluster Plant Developmental Biology, Wageningen University & Research, Droevendaalsesteeg 1, Wageningen 6708PB, The Netherlands; Cell and Developmental Biology, Cluster Plant Developmental Biology, Wageningen University & Research, Droevendaalsesteeg 1, Wageningen 6708PB, The Netherlands; Cell and Developmental Biology, Cluster Plant Developmental Biology, Wageningen University & Research, Droevendaalsesteeg 1, Wageningen 6708PB, The Netherlands; Cell and Developmental Biology, Cluster Plant Developmental Biology, Wageningen University & Research, Droevendaalsesteeg 1, Wageningen 6708PB, The Netherlands; Cell and Developmental Biology, Cluster Plant Developmental Biology, Wageningen University & Research, Droevendaalsesteeg 1, Wageningen 6708PB, The Netherlands; Keygene N.V., Agro Business Park 90, Wageningen 6708 PW, The Netherlands; Keygene N.V., Agro Business Park 90, Wageningen 6708 PW, The Netherlands; Keygene N.V., Agro Business Park 90, Wageningen 6708 PW, The Netherlands; Keygene N.V., Agro Business Park 90, Wageningen 6708 PW, The Netherlands; Keygene N.V., Agro Business Park 90, Wageningen 6708 PW, The Netherlands; Cell and Developmental Biology, Cluster Plant Developmental Biology, Wageningen University & Research, Droevendaalsesteeg 1, Wageningen 6708PB, The Netherlands; Bioinformatics Group, Wageningen University & Research, Droevendaalsesteeg 1, Wageningen 6708PB, The Netherlands; Molecular Biology, Cluster Plant Developmental Biology, Wageningen University & Research, Droevendaalsesteeg 1, Wageningen 6708PB, The Netherlands; Cell and Developmental Biology, Cluster Plant Developmental Biology, Wageningen University & Research, Droevendaalsesteeg 1, Wageningen 6708PB, The Netherlands

## Abstract

Plants have a remarkable regenerative capacity, but this varies widely among species and tissue types. Important crop cultivars show regenerative recalcitrance, which is a major obstacle for the application of modern plant propagation and breeding techniques. Regeneration generally involves empirically determined tissue culture methods that are based on the principle of inducing totipotency. Cells are first persuaded to change fate toward root stem cell-like identity and then are reprogrammed to acquire shoot fate. Alternatively, pluri- or totipotent cells can lead to the formation of a complete plantlet through somatic embryogenesis. We applied our knowledge of root stem cell niche biology to directly use the implicated stem cell factors, including *RETINOBLASTOMA* (*RBR*), *SCARECROW* (*SCR*), *SHORT ROOT* (*SHR*), and members of the *AINTEGUMENTA-LIKE/PLETHORA* (*AIL/PLT*) and *WUSCHEL-related homeobox* (*WOX*) gene families, as a tool to induce regeneration in a way similar to the principle of induced pluripotent stem cells in the animal field. We show that stem cell factors synergistically induce regeneration involving the somatic embryogenesis pathway and can break recalcitrance in Arabidopsis (*Arabidopsis thaliana*) and pepper (*Capsicum annuum*).

## Teaser

Stem cell transcription factors synergistically induce regeneration involving somatic embryogenesis and can break recalcitrance in Arabidopsis and pepper without added phytohormones.

## Introduction

Animals and plants can effectively withstand and recover from physical damage and environmental challenges. This may involve direct replenishment of damaged cells through the activity of adult stem cells, or an indirect strategy through dedifferentiation of somatic cells, which revert to a stem cell-like state before redifferentiating and regenerating new tissues or organs (Birnbaum and Alvarado 2008; Ince and Sugimoto 2023).

In plants, hormonal treatments can induce plant regeneration, a process that plays an important role in species preservation, breeding, and genetic improvement of crops. However, despite recent progress, recalcitrance to hormone-based regeneration is observed for many economically important species and genotypes, representing a serious bottleneck. The necessity to determine optimal hormonal and media compositions for each species, cultivars thereof, and even explants, can be laborious and is, in many cases, unsuccessful. To circumvent these problems, a gene-based, genotype-independent, and preferably hormone-free method for the acquisition of organogenic competence could be very useful.

Plant hormones can also induce totipotency in somatic cells by a process called somatic embryogenesis, which leads to the formation of an entire clonal plantlet. In practice, this is most often triggered by the application of the synthetic auxin derivative 2,4-D, which, in combination with the activation of endogenous auxin biosynthesis and the accumulation of lipids, is essential for the transition from a vegetative to embryonic state (Zhang et al. 2024). Such a switch can also be induced by the constitutive overexpression of somatic embryogenesis-related transcription factors such as the AINTEGUMENTA-LIKE/PLETHORA (AIL/PLT) member BABY BOOM (BBM), NF-YB and B3-AFL members LEAFY COTYLEDON (LEC) 1 and 2, or the homeobox containing WUSCHEL (WUS), in combination with additional hormone supply (Lotan et al. 1998; Stone et al. 2001; Zuo et al. 2002; Boutilier et al. 2002; Gaj et al. 2005). Other examples of regulators shown to improve the efficiency of genetic transformation and/or regeneration, usually together with existing phytohormone regimes, are PLETHORA5 (PLT5) (Lian et al. 2022; Wan et al. 2023), or combinations of WUS with BBM (Lowe et al. 2018) and GROWTH-REGULATING FACTOR with GRF-INTERACTING FACTOR (GRF-GIF) (Feng et al. 2021; Luo and Palmgren 2021; Bull et al. 2023).

The growing knowledge on these so-called developmental regulator genes involved in meristem development and somatic embryogenesis has led to their successful application in regeneration studies, but it is not yet clear why certain combinations of factors are successful and others are not. On the one hand, recent findings in rice that *OsBBM1* and *OsWOX9A* become coexpressed in the fertilized zygote through paternal protein transmission to initiate the development of the embryo (Ren et al. 2024) can be interpreted as a sign that zygote-specific transcription factors need to be brought together to confer pluripotency. On the other hand, both *BBM* and *WUS* are members of a large set of equivalent meristematic stem cell factors whose functions are interchangeable (Sarkar et al. 2007; Kerstens et al. 2024). Given that members of these families are coexpressed in stem cell niche regions of divergent plant lineages outside the seed plants (Zhang et al. 2017; Dipp-Álvarez and Cruz-Ramírez 2019; Motte et al. 2020; Liu et al. 2023; Fu et al. 2024), an alternative explanation is that the generic induction of stem cell niches is the key event in conferring pluripotency.

Indeed, pluripotent cells capable of organogenesis after wounding/and or hormone treatment acquire root stem cell-like identity, regardless of tissue origin (Sugimoto et al. 2010). For example, wounding triggers regeneration by activating cytokinin biosynthesis and reprogramming regulators such as WOUND INDUCED DEDIFFERENTIATION (WIND) factors and PLT3/5/7 to reactivate cell proliferation (Iwase et al. 2011; Ikeuchi et al. 2017). Phytohormone-induced regeneration, initiated by auxin-rich callus-inducing media (CIM), activates the lateral root primordium initiation program in pericycle(-like) cells (Sugimoto et al. 2010; Yin et al. 2024). Some primordia-like cells dedifferentiate to pluripotent QC-like cells in the so-called callus middle cell layer, requiring expression of *PLT1/2*, *WUSCHEL-related homeobox* (*WOX*)*5/7*, and *SCARECROW* (*SCR*) and SHORT ROOT (SHR) protein (Zhai and Xu 2021; Zhai et al. 2023). *PLT1/2*, *WOX5/7*, and *SCR* genes and the SHR protein are highly expressed there, which is required for a pluripotent status. PLT3/5/7 promote the expression of *PLT1/2* (Kareem et al. 2015), which is a prerequisite for the activation of *WUS* in the middle cell layer on cytokinin-rich shoot-inducing media (SIM) and subsequent shoot outgrowth (Zhai and Xu 2021). In agreement, Stages VI and VII lateral root primordia, where these genes are activated, can be directly converted to shoot primordia by cytokinin application only (Rosspopoff et al. 2017). Additionally, transcriptome studies of LEC2-expressing cells isolated from embryogenic callus revealed that root identity markers were expressed prior to the activation of shoot apical meristem (SAM) marker genes (Magnani et al. 2017). Collectively, these findings point toward the root stem cell niche as the key to regeneration competence.

In the *Arabidopsis thaliana* (Arabidopsis) root meristem, the stem cell niche is composed of infrequently dividing organizer cells of the quiescent center (QC), which is surrounded by mitotically active stem cells of the different tissues that make up the root (Dolan et al. 1993). Stem cell niche identity and maintenance is regulated by 2 pathways, involving the GRAS transcription factors SHR and SCR and the AIL/PLT family transcription factors (Benfey et al. 1993; Scheres et al. 1995; Di Laurenzio et al. 1996; Helariutta et al. 2000; Nakajima et al. 2001; Aida et al. 2004; Cui et al. 2007; Santuari et al. 2016).

The ability to reprogram somatic cells to a stem cell-like state through the ectopic expression of transcription factors was demonstrated initially in mice. Starting from a large set of candidate factors associated with pluripotency, a core set of 4 transcription factors was distilled, sufficient to induce embryonic-like pluripotent stem cells from mouse fibroblast cells when overexpressed (Takahashi and Yamanaka 2006). Given that cells involved in plant regeneration transit through a root stem cell-like state, we took a similar approach to induce regeneration-competent cells in the absence of exogenously added phytohormones. In this study, we reveal the potential of pluripotency-related transcription factors to induce organogenic competence and somatic embryogenesis in multiple plant species belonging to distantly related families.

## Results

### Selecting a core set of pluripotency-inducing transcription factors

A key intermediate step in hormone-induced in vitro regeneration is the acquisition of organogenic competence while passing through a root primordium and QC-like cell fate (Sugimoto et al. 2010; Zhai and Xu 2021; Yin et al. 2024). In the Arabidopsis (*A. thaliana*) root, complexes of SHR/SCR and PLT, together with additional binding partners, regulate the expression of *WOX5* in the organizer to maintain surrounding stem cells (Shimotohno et al. 2018). In addition, SCR interacts with the RETINOBLASTOMA-RELATED (RBR) protein to control QC division and the rate at which the surrounding stem cells get replenished (Cruz-Ramírez et al. 2013). Together, these factors orchestrate the intricate regulation of the primary and lateral root stem cell niches. In an attempt to bypass the hormone requirement to kick-start regeneration, we aimed to directly induce the competent stem cell-like identity. Therefore, we selected 2 sets of genes, henceforth referred to as the “dedifferentiation (DEDIF) genes” and the “stem cell niche (SCN) genes.” For the DEDIF set, *WIND1* and *RBR* were selected. Overexpression of *WIND1* was shown to induce somatic cell dedifferentiation, and a 2-step expression of *WIND1*- and *LEC2*-induced somatic embryogenesis in the absence of phytohormones in Arabidopsis (Iwase et al. 2011, 2015). The reduction of RBR leads to an increased amount of stem cells through the differentiation of columella cells (Wildwater et al. 2005). For the SCN set, we selected the *PLT1*, *PLT4/BBM*, *PLT5*, *SHR*, *SCR*, and *WOX5* genes because of their involvement in the specification and maintenance of the root stem cell niche (Supplementary Table S1).

**Figure 1.**
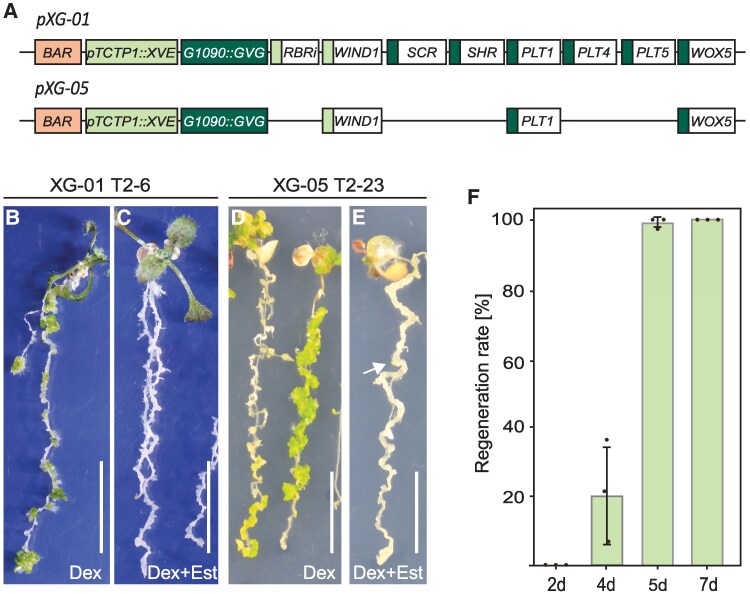
Overexpression of stem cell niche factors induces regeneration in the absence of exogenous phytohormones. **A)** Schematic overview of inducible expression vectors *pXG-01* and *pXG-05*. **B)** Induced overexpression of the full *SCN* gene set with 10 *µ*M Dex in the XG-01 line led to shoot regeneration from root tissue. **C)** Simultaneous induction of *DeDIF* and *SCN* genes on 10 *µ*M Dex and 10 *µ*M Est led to abundant callus formation. **D)** Induced overexpression of *PLT1* and *WOX5* in the XG-05 line was the minimal requirement for efficient shoot regeneration. **E)** Simultaneous induced overexpression of *WIND1*, *PLT1*, and *WOX5* resulted in abundant cell proliferation, with occasional shoot regeneration (arrow). Seedlings in **B**, **C**, **D**, and **E** were imaged at 14 dpi. **F)** Quantification of regeneration response after transient induction of *PLT1* and *WOX5* for 2, 4, 5, and 7 d. Regeneration was scored as the presence of shoots forming from the root at 21 dpi. The bars represent the mean over 3 independent replicate experiments ± SD, with *N* > 45 analyzed plants per timepoint. Scale bar = 1 cm.

To be able to regulate the timely expression of the selected DEDIF and SCN gene sets in Arabidopsis, we designed inducible constructs. In these constructs, we employed chemically inducible transactivation systems, whereby a synthetic transcription factor (transactivator) drives a set of transgenes of interest under control of a transactivator-specific target promoter (reviewed in Moore et al. 2006). We combined 3 of these systems: the ethanol-inducible AlcR/*AlcA* system (Caddick et al. 1998), the β-Estradiol (Est)-inducible XVE/*LexA* system (Zuo et al. 2000), and the Dexamethasone (Dex)-inducible GVG/*UAS* system (Aoyama and Chua 1997). The steroid-controlled components of the latter 2 systems have been previously combined to sequentially induce expression of different transgenes (Iwase et al. 2015). Using the Golden Gate cloning method, we initially created construct *pXG-01* (Fig. 1A). In this construct, the DEDIF genes *WIND1* and *amiGO-RBR* (here referred to as RBRi) are driven from the *LexA* promoter that is controlled by XVE. The root SCN genes *SHR*, *SCR*, *PLT1*, *BBM*, *PLT5*, and *WOX5* are driven from the *UAS* promoter that is controlled by GVG. To drive the expression of *XVE* and *GVG*, we opted for 2 different reported constitutive promoters: *XVE* expression is driven from the *TCTP1* promoter (Han et al. 2015), and *GVG* expression is driven from the *G1090* promoter (Ishige et al. 1999). The AlcR/*AlcA* system is not used in the current setup.

### Induction of root stemness-associated genes results in regeneration

We introduced the all-factor construct *pXG-01* in Arabidopsis ecotype Col-0 via floral dip and selected 11 T1 plants based on phosphinothricin (PPT)-resistance encoded by the *BAR* gene of the construct. PPT-resistant T2 seedlings from each line were transferred 7 d postgermination to a medium containing the inducer chemical for 2 wk. Mock treatment of transgenic XG-01 seedlings with DMSO did not appear different from WT Col-0 seedlings, suggesting no leakiness of the conditionally expressed transgenes. Induction of the root SCN gene set on medium containing 10 *μ*M Dex generally resulted in seedling growth arrest. In 4 of 11 lines, early regeneration events of green embryo- and shoot-like tissues were observed on the root (Fig. 1B, Supplementary Fig. S1A, Supplementary Table S2), which could develop into plantlets when explanted, indicating complete regeneration. We then tested these regenerating lines for the simultaneous induction of DEDIF and root SCN gene sets by exposing them to 10 *μ*M Est and Dex. We observed that the seedling primary root developed excessive callus-like tissue, and some of the lines developed green embryo- and shoot-like structures (Fig. 1C, Supplementary Fig. S1A, Supplementary Table S2). When inducing only the DEDIF genes with Est in these lines, we observed abnormal lateral root development that resulted in short, thick callus-like structures, but regeneration toward embryo- and shoot-like structures did not occur (Supplementary Fig. S1A).

Thus, we were able to induce regeneration from the root using a combination of stem cell-related genes in the absence of phytohormone application.

### Combined induction of *PLT* and *WOX* is sufficient for regeneration from seedling roots

Finding that induction of the SCN gene set alone was sufficient for complete regeneration, we next tested which of these genes are minimally required for regeneration. We created additional constructs *pXG-02* through *pXG-07* by omission of one or more transcriptional units (Fig. 1A, Supplementary Fig. S1B) and again scored them for early regeneration events of embryo- and shoot-like structures. In particular, we reasoned that a combination of a *PLT* gene with *WOX5* (*pXG-05)* may enhance the previously observed limited shoot regeneration effect of only *WOX5* or *WUS* overexpression (Zuo et al. 2002; Rashid and Kyo 2009; Zhai and Xu 2021). As control, we tested seedlings harboring *35S* promoter-driven *WOX5-GR* (Sarkar et al. 2007) and confirmed that overexpression of only *WOX5* resulted in shoot regeneration from root tips upon Dex induction (Supplementary Fig. S2A). In case of seedlings harboring *35S::PLT1-GR* (Santuari et al. 2016), we only observed cell proliferation at 14 d postinduction (dpi) (Supplementary Fig. S2B).

We scored Arabidopsis T2 lines harboring *pXG-02* through *pXG-07* constructs for induced phenotypes at 14 dpi as described above. For each construct, we examined at least 10 independent T2 lines (Supplementary Table S2). Because a regeneration response was not observed upon induction of only the DEDIF gene set in XG-01 lines, we induced either the SCN gene set alone or in combination with the DEDIF gene(s). Growth of primary root, lateral roots, and shoots was arrested in all lines, an effect that likely resulted from overexpression of *PLT* genes (Santuari et al. 2016). Early regeneration events were only observed in Dex-induced XG-05 (*PLT1/WOX5*) lines (Fig. 1, D and E, Supplementary Fig. S1C, Supplementary Table S2). Inducing the selected SCN genes except *WOX5* (XG-07) failed to form such embryo- and shoot-like structures, thereby highlighting the importance of *WOX5* in this process.

We tested whether a sequential induction of *WIND1* followed by *PLT1/WOX5* could improve the regeneration efficiency. Therefore, we induced *WIND1* for 1, 2, 4, or 7 d by placing seedlings on media with Est, before transferring them to Dex media for *PLT1/WOX5* induction for another 14 d. We did not observe enhanced regeneration in these experiments compared to only Dex-induced regeneration (Supplementary Fig. S1D).

We then asked if a short period of transgene expression is sufficient to complete the regeneration response. We induced *PLT1/WOX5* in 7-d-old XG-05 seedlings by transferring them to Dex media for 2, 4, 5, and 7 d before transferring them back to medium lacking inducer. At 21 d after the start of the experiment, we found that 4 d of induction of *PLT1*/*WOX5* resulted in shoot regeneration events in around 20% of the seedlings on average (Fig. 1F, Supplementary Fig. S2, C to F), albeit restricted to 1 or 2 events per seedling (Fig. 1F, Supplementary Fig. S2D). Shorter induction for 2 d resulted in growth arrest of primary roots, but regeneration events were not observed (Fig. 1F, Supplementary Fig. S2C). In contrast, transient *PLT1/WOX5* induction for 5 or 7 d led to abundant shoot regeneration along the root in nearly all seedlings (Fig. 1F, Supplementary Fig. S2, E and F). The regeneration events observed after transient *PLT1*/*WOX5* induction suggest that the regeneration process gains autonomy once a tipping point is reached, becoming independent of transgene expression.

To determine whether the ability to induce regeneration from somatic tissue reflects a broader role for the combined action of AIL/PLT and WOX family transcription factors, we generated construct *pXG-08* (Supplementary Fig. S1B) and induced *BBM* together with *WUS* in the resulting transgenic lines. We found that this combination also efficiently induced early regeneration events from the root at 14 dpi (Supplementary Fig. S1C, Supplementary Table S2). We concluded that efficient induction of regeneration from the seedling root can be achieved by the combined ectopic expression of an *AIL/PLT* and *WOX* family member.

### *PLT1/WOX5*-induced proliferation differs with tissue type and developmental stage

**Figure 2.**
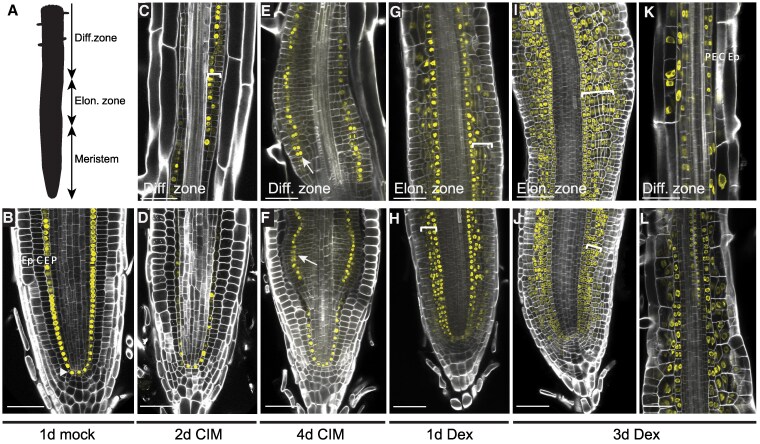
Proliferation and competent tissue formation induced by phytohormones versus PLT1/WOX5. **A)** Schematic view of developmental zones in the root. **B)** pSCR::SCR-YFP expression marking the QC (arrowhead) and endodermis of mock-induced XG-05 seedlings. **C)** Differentiation zone ∼1 cm from the tip at 2 dpi with CIM, showing the onset of callus formation from the pericycle (brackets), coinciding with expression of pSCR::SCR-YFP in the dividing pericycle cells. **D)** Root tip at 2 dpi with CIM showing little effect and maintenance of SCR-YFP accumulation in the endodermis. **E)** and **F)** Differentiation zone at ∼1, 5 cm **(E)** from the root tip **(F)** at 4 dpi with CIM, showing callus formation. Expression of pSCR::SCR-YFP was observed in 1 to 2 tissue layers, correlating to the callus middle cell layer (arrows). **G)** and **H)** Elongation zone **(G)** and root tip **(H)** of XG-05 seedling at 1 dpi of *PLT1/WOX5*, showing ectopic periclinal cell divisions in the cortex (brackets) and epidermis. Accumulation of SCR-YFP was observed mainly in cortex-derived cells. **I)** to **L)** XG-05 seedling elongation zone **(I)**, root meristem **(J)**, and differentiation zone at ∼ 3 mm **(K)** and at ∼1 mm **(L)** from tip at 3 dpi of *PLT1/WOX5*. In the elongation zone and meristem, periclinal cell divisions were observed in cortex (brackets) and epidermis, with SCR-YFP accumulation mainly in cortex-derived cells **(I**, **J)**. Higher up in the differentiation zone, periclinal cell divisions mostly occurred in the pericycle, accompanied by SCR-YFP accumulation **(K)**. At the start of the differentiation zone, correlating with initiation of root hair formation, an increase in periclinal cell divisions in the endodermis and pericycle was observed with accompanying SCR-YFP accumulation **(L)**. All images are of XG-05 seedling roots harbouring pSCR::SCR-YFP. Scale bar = 50 *μ*m. P, pericycle; E, endodermis; C, cortex; Ep, epidermis.

Similar to hormone-induced regeneration, the regeneration induced by overexpression of *PLT1/WOX5* appeared to proceed via an intermediary callus stage. To compare and visualize the origin of the callus-like tissue induced by phytohormones or by *PLT1/WOX5*, we crossed XG-05 plants to a reporter line carrying the *pSCR::SCR-YFP* translational fusion construct. SCR-YFP accumulates in the endodermis in WT roots (Di Laurenzio et al. 1996; Fig. 2, A and B) and is a marker for the pluripotent middle cell layer of phytohormone-induced callus (Zhai and Xu 2021). We induced callus for 4 d in F2 seedlings with either phytohormone induction (CIM) or by *PLT1/WOX5* induction and imaged the events over time using a confocal microscope.

In agreement with earlier findings (Atta et al. 2009; Zhai and Xu 2021; Zhai et al. 2023), phytohormone-induced proliferation appeared to develop from the pericycle in both the meristem and differentiation zone (Fig. 2, A, C, and D, bracket), and expression of SCR-YFP was limited to a 1 to 2 cell layer in the callus (Fig. 2, E and F, arrow). However, in Dex-induced *PLT1/WOX5* roots at 1 dpi, periclinal cell divisions were mainly observed in the cortex and epidermis in the meristem and elongation zone (Fig. 2, G and H, bracket). At 3 dpi, the number of epidermal and cortical cell divisions in the meristem and elongation zone had strongly increased, which led to severe thickening of the root tip (Fig. 2, I and J). Particularly, the cortex formed periclinal clonally related files of cells that now expressed SCR-YFP (Fig. 2, I and J, brackets). Around the beginning of the differentiation zone, we observed a decrease in cell divisions in the cortex and epidermis, and an increase thereof in the endodermis and pericycle (Fig. 2K). Higher up in the differentiation zone, at ∼3 mm from the tip, periclinal cell divisions were almost exclusive to the pericycle, coinciding with accumulation of SCR-YFP (Fig. 2L). At all timepoints, accumulation of SCR-YFP was observed in the majority of dividing tissue layers upon *PLT1/WOX5* induction, while being excluded from the outermost cells and the vasculature in the root meristem (Fig. 2, G to L).

Together, these results show that cell division patterns induced by *PLT1/WOX5* differ from those induced by phytohormones and possibly result in additional callus middle-layer-like cells, as indicated by the excessive SCR-YFP accumulation.

### Timeline of transgene-induced regeneration

To determine a timeline for the root stem cell factor-induced regeneration, we followed phenotype development of XG-05 (*WIND1*, *PLT1*, and *WOX5*) and XG-01 (all factors) seedlings over a period of 42 d after transfer to induction plates with either Dex or Est + Dex.

**Figure 3.**
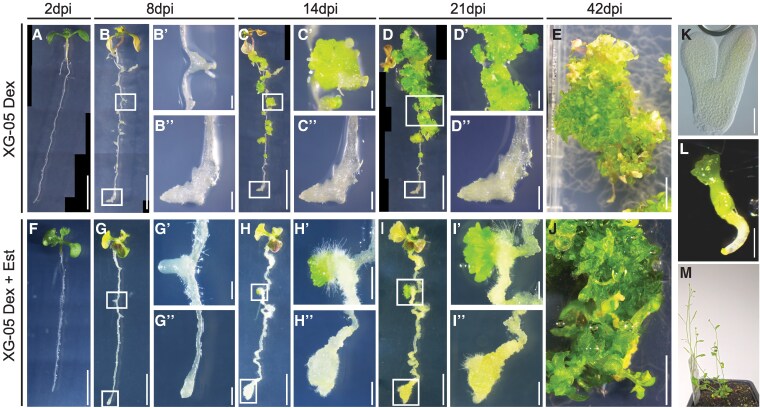
Time series of transgene-induced regeneration in XG-05 plants. **A)** to **E)** Representative images of XG-05 seedlings upon *PLT1/WOX5* induction with 10 *μ*M Dex, with close ups at 8 **(B-B″)**, 14 **(C-C″)** and 21 dpi **(D-D″)** of followed lateral root primordia and root tip. **F)** to **J)** Representative images of XG-05 seedlings upon *WIND1/PLT1/WOX5* induction with 10 *μ*M Dex and Est, with close ups of followed lateral root primordia and root tip at 8 **(G-G″)**, 14 **(H-H″)**, and 21 dpi **(I-I″)**. **K)** and **L)** Somatic embryo-like structures dissected at different developmental stages. **M)** Dissected tissue regenerated from Dex induced XG-05 seedling grown on soil develops into viable plants producing flowers and seeds. The image shown in Panels **A**, **B**, **C**, **D**, **F**, **G**, **H**, **I**, and **J** is a composite image. Scale bar in **A** to **J** = 1 cm, in **B′-D′**, **B″-D″, G′-I′, and G″-I″** = 1 mm and in **K** and **L** = 0.5 mm.

In the induced seedlings overexpressing *PLT1/WOX5* (XG-05) or the complete SCN gene set (XG_01), the first macroscopic signs of regeneration became apparent between 2 and 8 dpi, where callus-like tissue had formed on the root. This was particularly obvious at the lateral root sites and the primary root tip (Fig. 3, A and B-B″, Supplementary Fig. S3, A and B-B″). At some sites, the proliferating tissue was already greening at 8 dpi (Supplementary Fig. S3B′). Regeneration along the root of structures resembling somatic embryos was obvious from 14 dpi onward for both XG-05 and XG-01 lines (Fig. 3, C, D, K, and L, Supplementary Fig. S3C-C″). Shoot structures, characterized by the formation of leaves with trichomes, developed at multiple locations by 21 dpi (Fig. 3D-D″, Supplementary Fig. S3D-D″), culminating in an abundance of regenerating shoots at 42 dpi (Fig. 3E, Supplementary Fig. S3E). Seedlings induced to overexpress SCN genes in combination with DEDIF genes also regenerated somatic embryos and shoots after 14 and 21 dpi, respectively, but these appeared to regenerate at a slower pace compared with SCN gene expression alone (Fig. 3, F, G-G″, H-H″, and I-I″, Supplementary Fig. S3, F, G-G″, H-H″, I-I″, and J).

Viable plants that generated seeds developed from shoot structures that were dissected and transferred to soil after 42 d of growth on the inducing medium, indicating complete regeneration (Fig. 3, E, J, and M).

### Time-course RNA sequencing indicates extensive transcriptome reprogramming

To characterize the transcriptomic framework underlying the regeneration process induced by the overexpression of SCN gene sets in Arabidopsis, we conducted a time-course RNA-seq experiment. Roots of homozygous XG-01 (all SCN genes) and XG-05 (*PLT1/WOX5*) lines were sampled at 4 h, 10 h, 1 d, 2 d, 3 d, 7 d, 10 d, and 14 d after SCN gene induction, with controls including mock treated roots for 4 and 10 h and a 4 h mock-treated XG-05 seedlings shoot sample.

**Figure 4.**
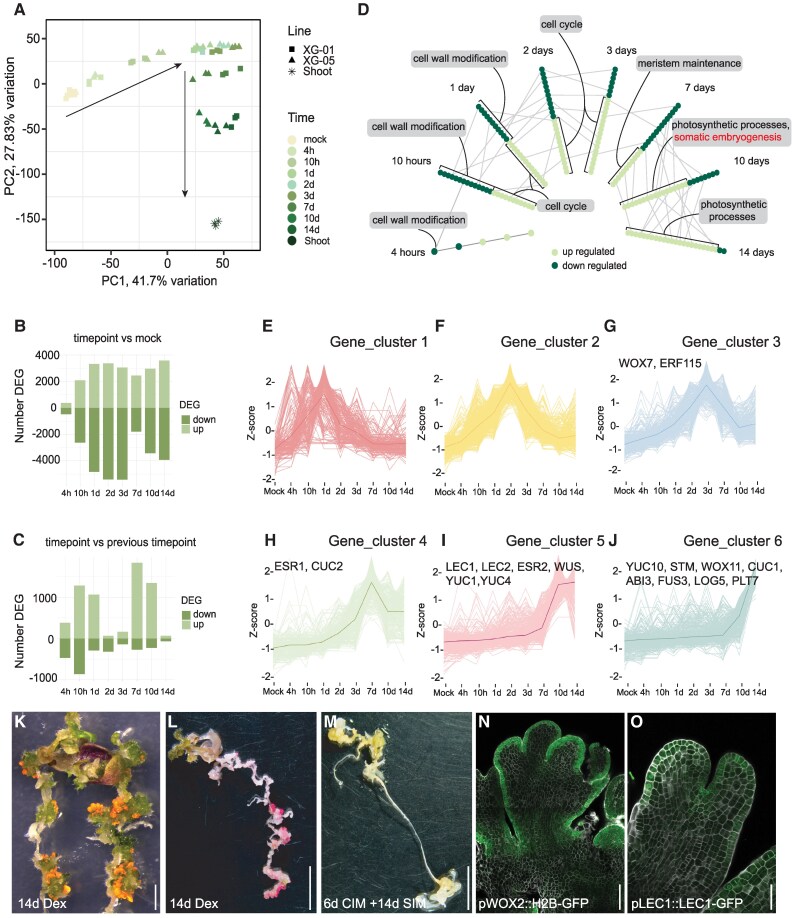
Differential gene expression analysis upon PLT1/WOX5-induced regeneration implicates the somatic embryogenesis pathway. **A)** Principal component gene expression analysis of the induced regeneration time-course experiments in XG-01 and XG-05 lines. Three biological replicates are represented by identical colors. Mock (roots) and shoot samples were from 4 h DMSO-treated seedlings. **B)** Number of DE genes upon *PLT1/WOX5* induction, per timepoint versus mock. **C)** Number of DE genes upon *PLT1/WOX5* induction compared to the previous timepoint. **D)** Gene set enrichment analysis (GSEA) of GO terms, with *P*_adj_-values <0.05, by enrichment score statistic as implemented in the fgsea R package, enriched in up- or downregulated genes at each timepoint compared to mock. Each dot represents a gene set of a GO term. Dark and light green dots represent downregulated (NES >1) and upregulated gene sets (NES < 1), respectively. GO-terms appearing at multiple timepoints are connected by lines. **E)** to **J)** Genes activated in XG-05 roots upon *PLT1/WOX5* induction with TPM <2 in mock and log2[FoldChange] > 2 at any timepoint compared to mock. Genes were grouped into 6 expression clusters. Thick lines represent average values. **K)** RFP fluorescence indicated embryo-like structures on an XG-05FR seedling root at 14 dpi of *PLT1/WOX5*. **L)** Sudan Red staining of an XG-05 seedling at 14 dpi of *PLT1/WOX5*, indicating regenerating somatic embryo-like structures. **M)** Sudan Red staining of XG-05 seedling root explant cultivated for 6 d on CIM, followed by 14 d SIM treatment. **N)** and **O)** Part of regenerating XG-05 seedling root at 11 dpi of *PLT1/WOX5*, showing emerging somatic embryo-like structures with epidermal *pWOX2::H2B-GFP*  **(N)** and pLEC1::LEC1-GFP **(O)** expression. The image shown in Panel **N** is a composite image. Scale bar = 5 mm **(K)**, 1 cm **(L, M)**, 250 *μ*m **(N)**, and 100 *μ*m **(O)**.

We performed principal component analysis (PCA) with the RNA-seq results of both XG-01 and XG-05 samples and included a shoot sample as an endpoint of development (Fig. 4A). The raw counts of all genes and TPM values of differentially expressed genes (DEGs) are listed in Supplementary Data S2. The first 2 components of the PCA show a high degree of similarity between the biological replicates and samples separated by time. There was no clear separation between the XG-01 and XG-05 lines at any timepoint, suggesting that both gene sets induce the same biological processes. We observed a developmental trajectory within the PCA, following a straight line in the first principal component (PC1, arrow) from the root mock samples up to the 3 dpi. This corresponded with the callus proliferation phenotype, which we observed in the first days after induction (Fig. 2, I to L). From the 3 to 14 d timepoints, the trajectory follows the second principal component (PC2, arrow) toward the shoot sample. The change in the direction observed in the PCA biplot agrees with the phenotypic changes we observed on these later timepoints, such as callus greening at 7 d, formation of somatic embryo-like structures at 14 d, and shoots at 21 d (Fig. 3C-C″, Supplementary Fig. S3C-C″). Since both time-course experiments showed the same behavior in the PCA plot, we decided to focus our analysis on the effects in the XG-05 line (*PLT1/WOX5* induction).

We compared all induction timepoints to the 4 h mock-treated root samples to identify DEGs. In total, 7,799 unique DEGs were identified across all timepoints. We discovered that, in addition to the upregulation of genes by the *PLT1/WOX5* overexpression, an even greater number of genes were downregulated in the first few timepoints (10 h to 3 d, Fig. 4B). To determine the gene activity during the time-course, we further compared the gene expression changes between consecutive timepoints. Interestingly, we found that a relatively small number of genes were differentially expressed between 2 d vs 1 d, 3 d vs 2 d, and between 14 d vs 10 d timepoints (Fig. 4C). Particularly, the comparisons between Days 2 and 3 suggest a transition phase, during which the callus stage of regeneration shifts toward somatic embryogenesis followed by shoot formation. This shift is reflected by the change in direction observed in the PCA biplot between Days 3 and 7 (Fig. 4A).

Gene set (GSEA) and Gene Ontology (GO) enrichment analysis of up- and downregulated genes indicated cell cycle reactivation followed by cell division as the initial response to upregulation of *PLT1*/*WOX5* at 10 h postinduction (hpi) (Fig. 4D, Supplementary Data S3). During the following days, cells keep on dividing intensely, resulting in abundant proliferation along the root of induced XG-05 (*PLT1*/*WOX5*) seedlings, which is again represented by the cell cycle as a main enriched GO term. Out of the 35 genes associated with the cell cycle (Menges et al. 2005), we found a total of 28 genes to be differentially expressed between 10 hpi and 3 dpi (Supplementary Fig. S4, Supplementary Data S2). The simultaneous downregulation of cell wall-related pathways (Fig. 4D) agrees with findings that adjustment of cell wall status is required for rapid cell divisions to occur in order for callus to be formed (Soni and Bacete 2023). Corresponding with the observed green somatic embryo-like structures appearing beyond 8 dpi, photosynthesis-related pathways are upregulated (Fig. 4D).

Investigating the expression pattern of published root tissue marker genes (Shahan et al. 2022, Supplementary Data S3), we observed that 38 out of 46 of these are downregulated over the time course of 1 to 3 d of *PLT1/WOX5* induction (Supplementary Fig. S5). Interestingly, genes correlated with QC generally show transient upregulation around 2 to 3 dpi, suggesting an intermediate competence related regeneration stage, before the change in developmental trajectory toward shoot fate observed in the PCA analysis (Supplementary Fig. S5 (boxed), Fig. 4A). Therefore, we analyzed expression of genes highly expressed in the middle layer of regenerative callus (Zhai and Xu 2021). We found a clear and transient upregulation of middle-layer genes at the 2 and 3 dpi timepoints of *PLT1/WOX5* induction (Supplementary Fig. S6), suggesting a window of regeneration competence at this time.

Together, these results are indicative of an extensive transcriptome reprogramming in response to *PLT1/WOX5* overexpression, which correlates with the establishment of regeneration competence.

### Cluster analysis implicates the somatic embryogenesis pathway in *PLT1/WOX5*-induced regeneration

To enhance the focus on regeneration, we selected genes with undetectable or very low transcript numbers in the mock treatment (TPM value <2), but strongly upregulated at any timepoint after Dex induction compared to mock (log2FoldChange >2 and *P*_adj_ < 0.05). We grouped these selected genes into clusters and found that 6 clusters represented the major distinctive temporal expression patterns best during *PLT1/WOX5*-induced regeneration.

Clusters 1 and 2 did not contain any known genes correlated to regeneration (Ikeuchi et al. 2019) nor gave any significantly enriched GO terms (Fig. 4, E and F, Supplementary Fig. S7A). Upregulated regeneration-related genes (Supplementary Data S3) were found in the remaining 4 clusters (Fig. 4, G to J). For instance, *WOX7*, the closest homolog of *WOX5*, and *ERF115*, a central regulator of root tip regeneration, were found in Cluster 3 and showed a peak expression at 3 dpi (Fig. 4G). Previous work demonstrated that *ERF115* overexpression resulted in *WIND1* upregulation (Heyman et al. 2016). Accordingly, we also find *WIND1* upregulation shortly after the peak of *ERF115* expression (Supplementary Data S2), suggesting that wound-induced tissue repair programs are involved in *PLT1/WOX5*-induced regeneration. This is consistent with the observation that the overexpression of *WIND1* did not have a beneficial effect on *PLT1/WOX5*-induced regeneration.

In Cluster 4, transcript levels peak at 7 dpi and are gradually downregulated thereafter. GO terms related to biotic stress response and response to stress-related hormones such as jasmonic acid (JA), salicylic acid (SA), and ethylene are enriched (Fig. 4H, Supplementary Fig. S7A), aligning with confirming reports that stress response pathways are involved in regeneration (Mira et al. 2016; Zhou et al. 2019; Bashir et al. 2022; Nautiyal et al. 2023). Included in Cluster 4 are the regeneration-related factors *CUC2* (Motte et al. 2011; Kareem et al. 2015) and *ESR1* (Iwase et al. 2017), which are both involved in the promotion of shoot formation during regeneration. Other key genes for shoot meristem formation, such as *WUS* and *ESR2*, were found in Cluster 5, where transcript levels peak at 10 dpi (Fig. 4I).

Interestingly, cluster and GSEA analysis both show that genes implicated in somatic embryogenesis, such as the *LAFL* genes (*LEC1*, *ABI3*, *FUS3*, and *LEC2*), were upregulated at 10 to 14 dpi (Clusters 5 and 6, Fig. 4, I and J, Supplementary Fig. S7B). The *LAFL* genes are generally involved in the maturation process of zygotic embryos, whereas ectopic *LEC1* and *LEC2* could even induce somatic embryo formation (Lotan et al. 1998; Stone et al. 2001; Gaj et al. 2005; Horstman et al. 2017). Furthermore, *OLEOSIN* (*OLEO*) genes, participating in lipid storage, were upregulated from Days 10 to 14 and grouped in Cluster 6 (Fig. 4J, Supplementary Fig. S7A). The accumulation of storage materials such as oil bodies is a distinctive property of Arabidopsis embryos (Miquel et al. 2014), which may also be used as a proxy of somatic embryogenesis (Stone et al. 2008; Vetrici et al. 2021; Zhang et al. 2024). Their importance was shown by an *oleo4* mutant that was defective in somatic embryogenesis (Gliwicka et al. 2012). Indeed, we found 18 out of 23 genes listed under the GO term somatic embryogenesis being upregulated during the time course of our experiment (Supplementary Fig. S7B), supporting that the *PLT1/WOX5*-induced regeneration process transits via somatic embryogenesis.

To provide some mechanistic insight into the role of PLT and WOX proteins during the regeneration process, we asked whether genes implicated in cell cycle, middle cell layer, and somatic embryogenesis (Supplementary Figs. S4, S6, and S7B) could be direct targets of these transcription factors. Since both PLT1/WOX5 and BBM/WUS were similarly able to induce regeneration via somatic embryogenesis, we utilized publicly available ChIP-seq data for BBM (Horstman et al. 2015) and WOX5 (Zhang et al. 2025). We observed that, whereas only one-third of the cell cycle-related genes can be bound, the vast majority of middle cell layer and somatic embryogenesis-related genes can be bound by BBM, WOX5, or both (Supplementary Fig. S7, C to E, Supplementary Data S3).

Together, our RNA-seq analyses indicate that the simultaneous overexpression of *PLT1* and *WOX5* triggers a series of cellular responses ranging from cell division to cell wall remodeling to stress induction, leading to callus formation and reprogramming, and implicates somatic embryogenesis as a major pathway of the induced regeneration.

### Expression of somatic embryogenesis markers during PLT1/WOX5-induced regeneration

To confirm the formation of somatic embryos, we set out to test the accumulation of oil bodies during the *PLT1/WOX5*-induced regeneration. First, we considered the FAST-R seed selection marker that consists of the *OLEO1* promoter driving expression of *OLEO1*, encoding the most abundant oleosin in maturing embryos, fused with *TagRFP* (Siloto et al. 2006; Shimada et al. 2010). XG-01FR (Col-0) and XG-05FR (Col-0) T2 lines, wherein the resistance cassette was replaced with the FAST-Red seed selection marker, that were induced to regenerate with *PLT1/WOX5* revealed FAST-R reporter fluorescence as early as 14 dpi (Fig. 4K). Second, we applied Sudan Red 7B staining for triacylglycerol (Bouyer et al. 2011) and observed clear staining upon *PLT1/WOX5*-induced regeneration (Fig. 4L). We then examined Sudan Red 7B staining upon phytohormone-induced regeneration of Col-0 seedlings at 21 d, and observed that staining was completely absent from regenerated tissues (Fig. 4M). In addition, we crossed somatic embryogenesis-related reporters *pWOX2::H2B-GFP* (Breuninger et al. 2008) and the *pLEC1::LEC1-GFP* (Gaj et al. 2005) in XG-05 seedlings, to visualize the fate switch to an embryo developmental program. We observed *pWOX2::H2B-GFP* and *LEC1::LEC1-GFP* expression mainly within the epidermis of the developing somatic embryo at 11 dpi (Fig. 4, N and O). These observations are in agreement with results reported for *WOX2* expression in 2,4-D induced somatic embryogenesis (Karami et al. 2023), and the *LEC1::LEC1-GFP* expression pattern observed in zygotic embryos at the torpedo stage (Song et al. 2021). By performing a time-course analysis, we observed *LEC1::LEC1-GFP* expression in a few cells as early as 4 d after *PLT1/WOX5* induction, becoming more abundantly expressed at sites of prospective somatic embryos over time (Supplementary Fig. S8, A to K).

Together, these histological observations confirmed the RNA-seq results that the intermediate stages in PLT1/WOX5-induced regeneration involve the somatic embryogenesis pathway.

### Induction of SCN transcription factors overcomes regenerative recalcitrance across Arabidopsis accessions

We wondered whether SCN genes could induce regeneration in Arabidopsis accessions that were reported as recalcitrant to phytohormone-induced regeneration. In previous work, recalcitrance to phytohormone-induced regeneration was ranked from very responsive to almost completely recalcitrant (Motte et al. 2014; Lardon et al. 2020). The *RPK1* gene was associated with the observed regeneration recalcitrance in a number of these accessions (Motte et al. 2014). We selected a set of responsive, recalcitrant, and intermediate accessions and transformed them with the *pXG-01FR* and *pXG-05FR* constructs (Supplementary Fig. S1B). Additionally, we transformed different allelic *rpk1* mutants (Col-0 background). We generated multiple transgenic T2 lines of the selected accessions, which were subsequently tested for SCN genes-mediated regeneration by transferring 7-d-old FAST-Red-positive seedlings to plates containing 10 *µ*M Dex. Regeneration was examined weekly up to 5 wk after induction, whereby a line was scored as responsive when one or more seedlings showed regeneration events ranging from embryo-like structures to the formation of shoots. We were able to regenerate all tested accessions as well as the *rpk1* mutants upon induction of the SCN gene sets with either construct (Supplementary Fig. S9). These results suggest that the intrinsic mechanisms activated by the induction of SCN genes can overcome phytohormone-based regeneration recalcitrance.

### Translation of the *PLT1/WOX5* regeneration system to crops

To investigate the applicability of *PLT1/WOX5*-induced regeneration in crop species, we used lettuce (*Lactuca sativa*), tomato (*Solanum lycopersicum*), and the highly recalcitrant pepper (*Capsicum annuum*).

For the lettuce *pLsXG-05* construct, we incorporated the syntenic lettuce orthologs of *WIND1*, *PLT1*, and *WOX5* (Supplementary Fig. S10, A to I, Supplementary Table S3), whereas for the tomato and pepper *pSlXG-05* construct, we used the Arabidopsis genes. Within the *pLsXG-05* construct, the *XVE* and *GVG* transactivator genes were driven from the *Petroselinum crispum* ubiquitin promoter (Plesch and Ebneth 2011), and an additional RUBY selection marker (He et al. 2020) was included for the visual identification of transgenic calli (Supplementary Fig. S1B). The generated *pSlXG-05* construct contained the *35S* CaMV promoter to facilitate expression of the *XVE* and *GVG* genes, and a GFP reporter for the identification of transgenic callus.

**Figure 5.**
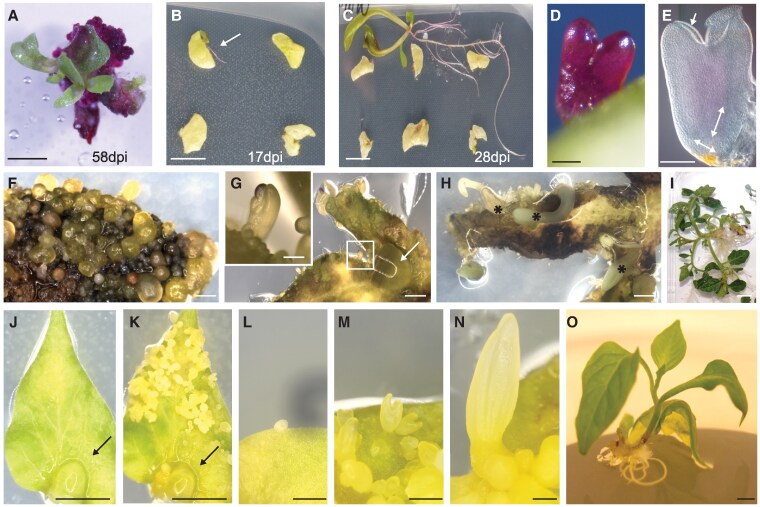
PLT1/WOX5 mediated regeneration in crops. **A)** Representative LsPLT1/LsWOX5-induced callus and regenerating shoots formed from a *pLsXG-05*-transformed lettuce explant. **B)** and **C)** Seedling regenerated from *pLsXG-05*-transformed and LsPLT1/LsWOX5-induced lettuce explant at 17 dpi **(B)** and 28 dpi **(C)**. **D)** and **E)** Embryo-like structure regenerated from *pLsXG-05*-transformed and LsPLT1/LsWOX5-induced lettuce explant at 14 dpi, imaged when still attached to the explant **(D)** and after dissection with DIC microscopy **(E)**. Arrow in **(E)** indicates L1 layer-like cell arrangement, double arrows indicate perpendicular cellular arrangement in vascular and root cap-like tissue. **F)** Transgenic SlXG-05 tomato cotyledon after 4 wk of *PLT1/WOX5* induction, showing abundant regeneration of globular-staged somatic embryo-like structures. **G)** Transgenic SlXG-05 tomato leaf showing efficient formation of somatic embryo-like structures 4 wk postinduction of *WIND1/PLT/WOX5* with a locally applied 10 *μ*M Dex and 1 *μ*M Est-loaded hydrobead (arrow). Inset shows a somatic embryo. **H)** Embryo development following local hydrobead application, resulting in the formation of harvestable cotyledonary stage somatic embryos (asterisks). **I)** Regenerated SlXG-05 tomato shoots 11 wk after induction. **J)** Local hydrobead (arrow) application on the first true leaf of a transgenic SlXG-05 pepper seedling. **K)** Strong induction of pepper somatic embryos 2 to 4 wk after hydrobead (arrow) application. **L)** to **N)** Examples of subsequent stages of pepper somatic embryo development following hydrobead application, from the inception of globular embryo **(L)** to the heart stage **(M)** to the late torpedo stage **(N)**. **O)** Harvested cotyledonary stage SlXG-05 pepper embryo grown out to a full-rooted plantlet. Scalebar = 1 cm **(A to C**, **I**, and **O)**, 250 *μ*m **(D**, **E**, and **L** to **N)**, 1 mm **(F** to **H)**, and 200 *μ*m (G inset) or 5 mm **(J** and **K)**.

For lettuce, we first tested whether induced overexpression of *LsPLT1/LsWOX5* could increase the efficiency of shoot regeneration following explant transformation in the presence of phytohormones. Therefore, *pLsXG-05* was introduced into the first leaf explants from 10-d-old lettuce (cv. Cobham Green) seedlings using Agrobacterium-mediated transformation. Cocultivation and subsequent shoot induction of these explants was performed on media supplemented with 0.1 *µ*M NAA and 0.1 *µ*M BAP, either with or without 10 *µ*M Dex to induce *LsPLT1/LsWOX5* expression, and the number of explants that formed shoots was determined after 28 d (Supplementary Fig. S10, J to L). In 2 independent experiments, we observed a significantly higher number of explants forming shoots on media containing phytohormones and Dex compared with only phytohormones (Supplementary Fig. S10L; 98% vs 77% and 66% vs 39%, respectively). This result is in agreement with other studies showing that overexpression of developmental regulators in the presence of phytohormones enhances shoot regeneration in both monocot and dicot crops, including wheat, cereals, maize, tomato, and melon (Lowe et al. 2016; Feng et al. 2021; Aregawi et al. 2022; Lian et al. 2022; Wang et al. 2022b). Next, we questioned whether *LsPLT1/LsWOX5* could induce regeneration in lettuce explants in the absence of added phytohormones. In 2 independent transformations, we observed shoot formation from ∼10% of explants (9/80 and 10/125) after a prolonged *LsPLT1/LsWOX5* induction (Fig. 5A). Surprisingly, we also observed the development of complete seedlings from explants (4/80 and 7/125, respectively) as early as 17 dpi (Fig. 5, B to E), suggesting regeneration via somatic embryogenesis. In line with this, we observed transgenic embryo-like structures developing from explants upon *LsPLT1/LsWOX5* induction in the absence of phytohormones at around 14 d after transformation (Fig. 5D). Dissection and subsequent microscopic analysis showed that some of these structures clearly displayed hallmarks of embryo patterning, such as central long, narrow apical-basal oriented cells, resembling vascular cells, ending at the basal pole in cells oriented perpendicular to these, together resembling a root pole (Fig. 5E, double arrows). At the apical pole, neatly arranged epidermal cells, resembling the protoderm, lined the outside of cotyledon-like structures (Fig. 5E, arrow). This is considerably shorter than the 6 wk typically required for the phytohormone-induced lettuce plant regeneration protocol, which also involves a separate rooting step. Together, these results indicate that the inducible expression of *LsPLT1/LsWOX5* effectuates the regeneration of both shoots and somatic embryos growing out into complete seedlings, following Agrobacterium-mediated explant transformation in lettuce in the absence of phytohormones.

The potential of induced *PLT1/WOX5* expression to promote regeneration in *Solanaceae* was tested by the introduction of the pSlXG-05 construct (Supplementary Fig. S1B) into tomato (cv. Moneyberg^+^) and pepper (cv. Maor) using *Agrobacterium*-mediated transformation. For tomato regeneration in the absence of phytohormones, 2 experiments were performed whereby *WIND1* was induced during the Agrobacterium-explant cocultivation step, after which the explants were transferred to *PLT1/WOX5*-inducing media. Callus and shoot regeneration were recorded 28 d after the start of the experiments. We observed that induction with *WIND1/PLT1/WOX5* resulted in hormone-independent shoot formation (Supplementary Table S4), albeit only in the absence of kanamycin selection. Stable transgenic SlXG-05 tomato lines were obtained through standard hormone-based regeneration and selection. We next tested the potential of *PLT1/WOX5* to induce regeneration on young tomato cotyledons and first true leaves in single-copy transgenic lines in the absence of phytohormones. Massive formation of globular structures across the surface was observed on hormone-free medium supplemented with 10 *µ*M Dex or Dex + Est (Fig. 5F). These globular embryo-like structures displayed severe growth arrest and did not develop further toward the cotyledonary stage. We reasoned that the observed growth inhibition may be attributed to growth competition or incomplete embryo formation associated with overall high *PLT1/WOX5* levels. To better regulate induction across the leaf surface, we locally applied inducive hydrobeads supplemented with 10 *µ*M Dex and 1 *µ*M Est, on the adaxial site of cotyledons or first true leaves (Fig. 5, J and G, arrow). This alternative induction method resulted in the formation of multiple cotyledonary embryos at reduced frequencies that could be nurtured into full plants (Fig. 5, H and I). Additional experiments with hydrobeads loaded with only 10 *µ*M Dex gave very similar results. Together, these results show that *PLT1/WOX5* induction is sufficient to induce shoot regeneration involving the somatic embryogenesis pathway in stable transgenic tomato lines in the absence of any phytohormones.

Pepper is considered a recalcitrant species, in which successful transformation and plant regeneration using phytohormones has been documented for only a few individual genotypes at very low frequencies (Lee et al. 2004; Heidmann et al. 2011). We tested the effect of *WIND1* and *PLT1/WOX5* induction in 2 independent pepper transformation experiments in combination with hormones (*n* = 860) but did not observe developing embryogenic structures nor shoot outgrowth. However, independent transgenic calli expressing GFP were obtained, and after prolonged cultivation on various phytohormone regimes, some of these transgenic calli produced leafy structures that lacked a functional shoot or root meristem. To obtain a stable transgenic SlXG-05 pepper line, induction of *WIND1* and *PLT1/WOX5* was performed directly on the leafy structure using inducive hydrobeads loaded with 1 *µ*M Est and 10 *µ*M Dex. Analogous to tomato, several cotyledonary stage embryos were formed, of which 2 were nurtured into rooted plantlets. The validated transgenic plants were transferred to the greenhouse for the next generation and homozygous T2 seed production. We then tested if *WIND1/PLT1/WOX5* overexpression was able to induce regeneration in the absence of phytohormones in these stable transgenic SlXG-05 pepper lines. Local application of inducive hydrobeads resulted in efficient somatic embryo formation 2 to 4 wk after induction (Fig. 5, J to N), and rooted plants were obtained within 11 wk (Fig. 5O). These results showed that pepper regeneration recalcitrance can be overcome by effectively triggering the somatic embryogenesis pathway through induced *WIND1*/*PLT1/WOX5* overexpression in the absence of phytohormones.

## Discussion

In this study, we adopted a strategy analogous to the discovery of induced pluripotent stem (iPS) cells in animals by combining the expression of key pluripotency factors from the root stem cell niche to enhance cellular competence. We demonstrated that these combined transcription factors induced efficient regeneration in the absence of phytohormones. Specifically, a minimal combination including a *PLT* and *WOX* member can induce regeneration in Arabidopsis, lettuce, tomato, and pepper. These results are in agreement with the reported stimulation of transformation in other species by the *BBM/WUS* combination, albeit mostly in the presence of phytohormones (Lowe et al. 2016, 2018; Hoerster et al. 2020; Xu et al. 2022; Wang et al. 2023; Sato et al. 2024). Furthermore, we demonstrated the power of stem cell factor-induced regeneration by breaking recalcitrance in transgenic Arabidopsis accessions and in pepper, again in the absence of phytohormones. Phenotypic, transcriptomic, and marker analyses revealed that *PLT1/WOX5*-mediated regeneration is accomplished (at least in part) through the somatic embryogenesis pathway. Together, we conclude from our rational-design stem cell factor screen that *PLT* and *WOX* genes, as members of conserved and coexpressed meristematic stem cell factor families, are key to the induction of pluripotent stem cell properties that are required for regeneration.

Furthermore, during the establishment of callus from phytohormone-treated explants, the establishment of a QC-like identity, marked by *SCR* expression within the middle cell layer of the callus, conveyed pluripotency (Zhai and Xu 2021). Here, we showed that combined ectopic *PLT1/WOX5* expression promoted the formation of additional middle-layer-like cells, as suggested by the excessive SCR-YFP accumulation. This result suggests that the *PLT* and *SHR/SCR* pathways do not function fully in parallel during the acquisition of pluripotency, in contrast to their largely parallel roles in root stem cell niche patterning. A potential explanation is that the high expression of PLT induces the expression of meristematic factors, including its direct targets JKD and SHR (Santuari et al. 2016), with SHR subsequently activating SCR expression. This interpretation aligns with *SHR* and *SCR* being dispensable from the regeneration-inducing constructs, further emphasizing *PLT*-mediated regulation as a key driver of pluripotency acquisition.

The induction of *WOX5* was essential to advance the regeneration process in the root of transgenic Arabidopsis lines. *WOX5* was previously found to regulate cell cycle progression and enhance cytokinin signaling, thereby promoting both pluripotency and de novo shoot regeneration (Lee et al. 2022; Yang et al. 2024). *WOX5* and its homolog *WUS* are functionally interchangeable in some contexts, and their overexpression can induce shoot and somatic embryo regeneration from the root tip (Zuo et al. 2002; Sarkar et al. 2007; Rashid and Kyo 2009; Ikeda et al. 2020). Recent studies also showed that *WOX5* overexpression alone can induce somatic embryogenesis in the absence of exogenous auxin from immature Arabidopsis embryos (Wójcik et al. 2025). In our system, induced *WOX5* overexpression in roots triggered regeneration from lateral root tips. This difference likely relates to tissue context, with embryonic tissue being more responsive compared with the more mature root tissues. The restriction of regeneration to lateral root tips upon *WOX5* overexpression suggests that in these regions, local *PLT* levels are sufficient to provide a permissive environment.

Our observations indicate that the combination of PLT and WOX transcription factors synergistically influences gene expression and physiology, resulting in pluripotency acquisition. Together with the following observations, this points to a broader and evolutionary-conserved mechanism underlying meristematic and embryonic potential: (1) the combined overexpression of *OsBBM1* and *OsWOX9A* in rice zygotes synergistically induces apomixis (Ren *et al.* 2024), (2) *PLT1/2* and *WOX5/7* are required for pluripotency acquisition in regenerative callus (Zhai and Xu 2021), (3) PLTs were shown to physically interact with WOX5 to regulate and maintain columella stem cells (Burkart et al. 2022), (4) *PLT* family members are interchangeable for somatic embryo initiation (Boutilier et al. 2002; Horstman et al. 2017; Kerstens et al. 2024), and (5) combinations of *PLT* and *WOX* genes are expressed in the stem cell regions of early diverged species (Liu et al. 2023; Fu et al. 2024). Based on these insights and our findings that the *BBM/WUS* combination also induces somatic embryos from mature roots, we propose that meristematic potential is not determined by a specific *PLT/WOX* combination, but rather that coexpression of any *PLT* with any *WOX* is sufficient to confer pluripotency. Particularly, the deep conservation of *PLT/WOX* coexpression suggests that this pluripotency factor combination represents an ancient, conserved mechanism to define growth regions that predates seed plants, which was adopted for seed plant embryogenesis. We can speculate that the combined PLT/WOX effect, as we describe here for the induction of regeneration, involves direct protein complex formation or results from cooperative binding to the promoter of genes, effectuating transcriptional synergy (Georges et al. 2010). The formation of a PLT–WOX complex may further promote the expression of genes involved in chromatin remodeling to allow for cell fate transitions. Once dedifferentiation is initiated, increased chromatin accessibility may allow PLTs to bind and activate LAFL genes, thereby triggering the somatic embryogenesis program. Indeed, the vast majority of middle cell layer and somatic embryogenesis-related genes implicated in the regeneration response can be bound by PLT/WOX clade members BBM, WOX5, or both. The observation that the bound genes are expressed at different stages during the regeneration process suggests that chromatin accessibility is a determining factor in their regulation.

In lettuce (Asteraceae), we observed whole primary transformant seedlings regenerating in the absence of phytohormones within a short time window compared with standard hormone procedures. The accelerated development into a full seedling appeared to involve somatic embryogenesis and may actually have been aided by the absence of phytohormones in the growth media, thereby quickly allowing the establishment of correct hormone gradients needed for tissue organization (Wong et al. 2023). For the recalcitrant pepper (Solanaceae), we were not able to improve primary transformant regeneration efficiency by *PLT1/WOX5* with or without combined phytohormone treatment. However, in both stable transgenic tomato and pepper plants, abundant *PLT1/WOX5*-mediated regeneration in the absence of added hormones could be induced. Therefore, the obtained regenerative transgenic tomato and pepper lines present a valuable tool for future studies into recalcitrance in crops. The hydrobead experiments in pepper included induction of *WIND1* in addition to *PLT1/WOX5*, and although none of the other species tested for regeneration induction indicated a possible synergistic role of *WIND1*, the role of such so-called dedifferentiation factors is worth further study. The underlying cause for pepper recalcitrance may be multifactorial, with low transfection and T-DNA integration rates as part of the limiting factors not related to regeneration per se. One way of circumventing these may be to apply alternative delivery/transformation methods to effectively introduce the *PLT1/WOX5* cassette (Bélanger et al. 2024). The particular combination of (additional) factors and their independent levels of induction may further influence their effect in various plant tissues, cultivars, or species. Another strategy to potentiate the effect of *PLT1/WOX5* on regeneration may be the fusion of these to a transcriptional enhancer or repressor domain (Ikeda et al. 2020; Sato et al. 2024).

Phytohormone-induced de novo shoot organogenesis is thought to happen through spontaneous self-organization of meristem progenitors rather than guidance by preexisting patterns (Gordon et al. 2007; Xu et al. 2021; Varapparambath et al. 2022). Asymmetries reflected by distinct gene expression patterns, cell geometry, and mechanical inconstancies within these progenitors may result in self-organization through multiple feedback loops (Mathew et al. 2024), followed by the formation of a functional shoot meristem. So far, little is known about the process of self-organization during somatic embryogenesis, and marker genes for somatic embryo progenitor cells have not been identified (Wang et al. 2022a). Nevertheless, the observation that *PLT1/WOX5*-induced regeneration required an induction time of 4 d, which correlated with the earliest observed timepoint of LEC1 accumulation in sporadic cells, suggests that these may constitute such progenitor cells. In addition, *PLT1/WOX5*-induced regeneration even proceeded under continuous induction, suggesting the involvement of epigenetic reprogramming to allow the cascade of gene expression changes to continue. Whether and when reprogramming of epigenetic marks, which set the stage for expression of embryogenic and shoot-promoting factors in phytohormone-induced regeneration (Wang et al. 2020; Wu et al. 2022), occurs in *PLT1/WOX5*-induced regeneration remains to be tested. Mapping chromatin state combined with time-course ChIP-seq during the PLT/WOX-induced regeneration may clarify the interdependency of the epigenome and transcriptome during this process and provide clues to the apparent self-organization. Single-cell technologies applied to *PLT1/WOX5*-induced regenerating tissues in the absence of exogenous hormonal cues may facilitate the identification of regeneration-specific genes and epigenetic marks, as hormonal treatments often induce additional, unrelated cellular responses that can obscure regeneration-specific processes.

As an overall conclusion, we demonstrated that induced expression of root stem cell factors can drive cellular differentiation toward organogenesis across several plant cultivars within the Brassicaceae, Asteraceae, and Solanaceae families, including the notoriously recalcitrant pepper species. A noteworthy achievement of this study is the complete bypass of external hormone application requirements for regeneration across multiple species, overcoming a common bottleneck in tissue culture protocols used for plant transformation and genomic engineering. Our work paves the way for further investigations toward epigenetic reprogramming and gene regulatory networks, to deepen our understanding of the regeneration process.

## Materials and methods

### Plant materials and growth conditions

Experiments performed with Arabidopsis (*A. thaliana)* were done with ecotype Col-0 unless stated otherwise. Seeds were surface sterilized by fumigation with chlorine gas (Lindsey et al. 2017) in a desiccator jar for 4 h. Sterilized seeds were stratified at 4 °C in the dark for at least 48 h. Seeds were plated on half-strength Murashige and Skoog (MS) germination medium (1/2GM) that included vitamins, 0.8% plant agar, 0.5 g/l MES pH 5.8, and 1% sucrose. The plates were placed near vertically in a 22 °C growth chamber under a 16 h-light/8 h-dark cycle. For marker analysis, we used the previously described lines: SCR::SCR-GFP (Gallagher et al. 2004), LEC1::LEC1-GFP (Li et al. 2014), and WOX2::H2B-GFP (Gooh et al. 2015).

For testing regeneration efficiency through our SCN gene set in different Arabidopsis ecotypes, we used the Heynh. accessions (N22660): Uod-7, CIBC-17, Sq-8, Tamm-2, RRS7, Ga-0, PNA-17, Fei-0, Nok-3, LP2-2, Bor-4, KZ-9, NFA10, and Yo-0. In addition, we tested the *rpk1-1*, *rpk1-2*, and *rpk1-5* mutants (Nodine et al. 2007).

Transgenic lines were grown for 7-d-old, before transferring seedlings to media containing 10 *μ*M Dex. Unless otherwise indicated, we followed phenotype development of the T3-6 and T3-23 line for XG-01 and XG-05, respectively, which displayed a strong induced regeneration response, and of which the T2 segregated the construct as a single locus based on PPT resistance. For the transient induction experiment, 7-d-old germinated XG-05 seedlings were transferred to 10 *μ*M Dex-containing medium and cultivated on it for 2, 4, 7, or 10 d, before their transfer back to 1/2GM medium without inducers and cultivated for 42 d.

### Genetic construct generation

Binary constructs used in this research were assembled using the Golden Gate cloning method (Engler et al. 2009, 2014). Supplementary Data table S1 lists the primers used to amplify the genetic fragments and schematically displays the parts used to assemble level-0 constructs, the plasmids used to assemble level-1, and the final level-2 Golden Gate constructs.

### Identification of lettuce homologs of *PLT1*, *WOX5*, and *WIND1*

Protein sequences and annotations of tomato (*S. lycopersicum*), lettuce (*L. sativa*), and Arabidopsis were obtained from Solgenomics (SL2.5), CoGe (cv. Salinas V8; ID 28333), and TAIR (Araport11), respectively. AP2, EREBP, and homeobox homologs were identified by scanning the proteomes for the presence of 1 AP2 domain (PF00847; EREBP family), more than 1 AP2 domain (AP2 family), or at least 1 homeodomain (PF00046; homeobox family) with HMMER v3.2.1. Hits were aligned with MAFFT v7 using the BLOSUM62 matrix, the FFT-NS-2 strategy, and a gap opening penalty of 1.0. The multiple sequence alignment was trimmed with trimAl v.1.1 using the -gt 0.7 and -cons 0.5 flags (Katoh and Standley 2013), then fed to IQ-Tree v1.6.10 for phylogenetic analysis, using automatic model finding and 1,000 ultrafast bootstraps (Minh et al. 2020). Clades were defined by the presence of Arabidopsis proteins. Syntenic connections between genes were defined as sharing at least 5 homologous genes in the 25 up and downstream genes with MCScanX (Wang et al. 2012) and parameters -k5s5m25. Trees were visualized with iTOL (Letunic and Bork 2021).

### Agrobacterium-mediated transformation

For Arabidopsis transformation, the binary constructs were transformed in Col-0 with *Agrobacterium tumefaciens* strain C58C1.pMP90 by floral dip (Koncz and Schell 1986; Clough and Bent 1998). Transgenic seedlings were screened for resistance to the selection marker PPT (Duchefa Biochemie) on a plate (20 *µ*g/ml) or by spraying on soil-grown seedlings (100 *μ*g/ml). In case of FAST-R marker incorporation, fluorescent T1 seeds were selected under a fluorescence binocular (Leica MZ16F) Homozygous insertion lines were selected only from T2 lines showing Mendelian segregation of resistance or marker expression.

For phytohormone regeneration of Arabidopsis, seedlings were grown for 7 d on ½ GM medium. Optionally, the shoot and hypocotyl were cutoff, and explants were placed on ½ GM supplemented with 22 *µ*M 2,4-D and 2 *µ*M Kinetin for 7 d in long day conditions, 16 h-light/8 h-dark cycle. Explants were then transferred to SIM media consisting of ½ GM media supplemented with 25 *µ*M isopentenyladenine base (iP) and 8,6 *µ*M indole-3-acetic acid for 14 d at a 16 h-light/8 h-dark cycle. Hormones were dissolved in DMSO and supplied to the medium after autoclaving.

For lettuce transformation, *L. sativa* (cv. “Cobham Green”) seeds were surface-sterilized for 1 min in 70% EtOH and 10 min in 10% bleach with Tween-20, before washing them 5 times with water. Seeds were plated on 1/2GM medium and 0.8% agar at 22 °C under a 16 h-light/8 h-dark cycle. Transformation was performed as described (Michelmore et al. 1987) with modifications. Binary vectors were transformed into Agrobacterium strain Agl-1 (Lazo et al. 1991). Transformed Agrobacteria were grown in MGL medium containing 20 mg/l rifampicin and 50 mg/l kanamycin for 2 d at 28 °C overnight in a shaking incubator (250 rpm). After 48 h incubation, 5 ml of the culture was added to 15 ml of TY medium (pH 5.5) containing the appropriate antibiotics and 200 *µ*M acetosyringone. The culture was incubated for 16 h at 28 °C at 250 rpm, before diluting the bacteria with TY medium and 200 *µ*M acetosyringone to an OD600 of ∼0.2. The first true leaves from 10- to 14-d-old lettuce seedlings were submerged in the Agrobacteria dilution and cut in half. After the tissue was soaked for 5 to 10 min, the remaining excess liquid was removed from the explants using filter paper, which were then placed on cocultivation medium consisting of Schenk and Hildebrandt (SH) basal medium with added MS vitamins, 30 g/l sucrose, 0.8% (Daishin) agar, and 200 *µ*M acetosyringone, pH 5.8. After 3 d, explants were transferred to fresh SH medium supplemented with 300 mg/l timentin and 100 mg/l kanamycin. For phytohormone-induced regeneration, 0.1 *µ*M 1-naphthaleneacetic acid (NAA) and 0.1 *µ*M 6-benzylaminopurine (BAP) were added to the medium, and for transgene induction, 10 *µ*M Dex or an equivalent amount of DMSO (mock) was added. After transformation, explants were cultured at 20 °C under a 16 h-light/8 h-dark cycle and transferred to fresh medium every 14 d.

For tomato transformation, Agrobacterium-mediated transformation was performed as described (Koornneef et al. 1986, 1987) with modifications. Tomato seeds were sterilized and germinated on 1/2MS10 medium for 10 d. Cotyledon explants from the seedlings were dissected and precultured for 24 h on cocultivation medium supplemented with 40 *µ*g/l acetosyringone. The explants were submerged in a suspension of Agrobacterium tumefaciens GV3101 carrying *pSlXG-05* grown overnight in LB medium containing 100 mg/l kanamycin and 25 mg/l rifampicin, and diluted to an OD600 range of 0.13 to 0.15. The explants were blotted dry and cocultivated for 2 d on plates of MS20 medium with 40 mg/l acetosyringone. The experimental treatment consisted of preinduction of the transcription factor set by the addition of 10 *µ*M Est during the cocultivation without the presence of any hormones. The control treatment consisted of the addition of 2 mg/l NAA and 1 mg/l BAP to the cocultivation medium. After cocultivation, the explants were transferred to MS20 medium with 200 mg/l cefotaxim and 100 mg/l vancomycin to suppress further Agrobacterium growth. In addition, this medium was supplemented with 10 *µ*M Dex to induce the transcription factor genes under the control of GVG (experimental treatment) or with 1 mg/l zeatin (control treatment). In this manner, the effect of the transactivated *PLT1* and *WOX5* genes was compared with the addition of plant growth regulators. The explants were cultivated at 25 °C and 3000 lux (16/8 h photoperiod) in a growth chamber. The explants were subcultured every 2 wk onto fresh medium. Single-copy insert lines were transferred to the greenhouse for seed production and subsequent homozygous T2 seed generation to be used in induction experiments. Induction experiments on stable transgenic tomato lines were performed with inducive hydrobeads. Hydrogel beads were generated by pipetting 10 *µ*l of a 1.6% alginate solution in an excess of 1% CaCl_2_ solution. Beads were harvested and equilibrated in liquid MS, including vitamins medium containing 20 g/l sucrose and 0.5 g/l MES (pH 5.8) for over an hour. Beads were subsequently equilibrated in an inducer solution containing the same medium supplemented with 1 *µ*M Est and/or 10 *µ*M Dex.

For pepper (cv. Maor), transformation was performed as described (Heidmann et al. 2011) with some modifications. Agrobacterium strain GV3101 carrying the *pSlXG-05* construct was grown with the appropriate antibiotics in 100 ml LB medium at 28 °C. During cocultivation with 10-d-old cotyledons, the medium included hormones only, or was supplemented with either 10 *µ*M Est or 10 *µ*M Dex. Following cocultivation, explants were transferred to selection medium consisting of cocultivation medium supplemented with 1 mg/l thidiazuron (TDZ; Murthy et al. 1998), 100 mg/l kanamycin sulfate, 500 mg/l cefotaxime, and 10 *µ*M Dex. The explants with transgenic callus expressing GFP were sub-cultured every month onto fresh medium until the formation of leaf-like structures (∼4 to 5 mo after transformation). To allow the generation of stable transgenic plants, selected green leaf-like structures were induced using inductive (Est/) Dex-loaded hydrogel beads to induce somatic embryo formation. Properly developed cotyledonary stage plantlets were transferred to noninductive conditions for further development and rooting. Validated diploid plants were transferred to the greenhouse for seed production and subsequent homozygous T2 seed generation for induction experiments using hydrobeads. The explants were cultivated at 25 °C and 3000 lux (16/8 h photoperiod) in a growth chamber.

### Seedling image capture and digital stitching

Pictures of whole seedlings and/or roots on agar plates were taken with either a mounted camera (Nikon D3100) or with a stereoscopic microscope (Nikon SMZ745T). Where required, pictures were digitally stitched using the ImageJ Grid/Collection stitching plugin to generate a single image (Preibisch et al. 2009).

### Analysis of time-course RNA-seq data

For time-course RNA-seq, homozygous transgenic XG-01 and XG-05 lines were first grown for 7 d on growth media on top of a nylon mesh as described (Birnbaum et al. 2003; Santuari et al. 2016). Subsequently, we transferred the nylon with the seedlings to 10 *µ*M Dex-supplemented media to transactivate the expression of the SCN genes and induce regeneration in time. Roots were cut below the hypocotyl and harvested at 4 h, 10 h, 1 d, 2 d, 3 d, 7 d, 10 d, and 14 d after induction; each timepoint comprised 3 biological replicates. As a control, we included mock-treated root samples, which were transferred to growth media supplemented with DMSO and harvested after 4 and 10 h. In addition, we collected the shoot tissues, including part of the hypocotyl, from the 4 h mock-treated seedlings. The NEBNext Ultra Directional RNA Library Prep Kit for Illumina (NEB #E7420S/L) was used for library preparation, and the RNA sequencing was performed using the Illumina NextSeq 500 platform, generating 150 bp single-end reads (GenomScan, Leiden, The Netherlands). After sequencing, the quality of the reads was assessed using the FastQC program (https://www.bioinformatics.babraham.ac.uk/projects/fastqc/). The RNA-seq reads were then mapped to the Arabidopsis genome (TAIR10) using HISAT2 (Kim et al. 2015) (see Supplementary Data S2 for mapping efficiency). The output SAM files were converted to sorted BAM files by SamTools (Li et al. 2009). Gene transcript abundance was determined using featureCounts (Liao et al. 2014) based on the Araport11 annotation. PCA was performed using the R package PCAtools v2.14.0 (Blighe and Lun, 2024) using the normalized expression data, including all genes. DEGs were detected using the R package DESeq v. 1.42.1 (Love et al. 2014). DEG at each timepoint compared to mock (4 hpi) or to the previous timepoint were identified based on the criteria: absolute log_2_ fold change >1 and *P*_adj_ < 0.05.

For cluster analysis, activated genes were selected using the combined criteria: TPM <2 at mock (4 hpi), log_2_ fold change >2, and *P*_adj_ < 0.05 in any of the timepoints. These genes were subjected to unsupervised clustering by the fuzzy c-means algorithm implemented in the Mfuzz package (Kumar and Futschik, 2007). The data were grouped into 6 clusters with a fuzziness parameter (m) estimated from the data. Genes were associated with a cluster with a minimum membership value (acore) of 0.5, meaning they are strongly associated with their assigned cluster.

### GSEA, GO-term, and ChIP-seq analysis

For gene set enrichment analysis (GSEA), up- and downregulated genes of each timepoint compared to mock (4 hpi) were ordered by multiplying log2fold change with log_10_ normalized *P*_adj_ as a ranked list of genes. GO terms for Biological Processes were used for enrichment analysis using the fgsea R package (Korotkevich et al. 2019). GO terms with *P*_adj_-values <0.05 were considered to be significantly enriched in upregulated (NES > 1) or downregulated (NES < −1) genes. *P*-values were determined based on the enrichment score statistic using the permutation-based method implemented in the fgsea R package. The significant GO terms per timepoint were visualized using the Python package Hiveplotlib (Krzywinski et al. 2012).

GO term analysis was performed using the enrichGO module from the clusterProfiler R package v.4.10.1. DEG of each timepoint compared to mock (4 hpi), with a log_2_ fold change >1 for upregulated genes and log_2_ fold change <−1 for downregulated genes, were selected for this analysis. The combined DEGs from all samples were used as the background gene set.

Overlaps between BBM (Horstman et al. 2015) and WOX5 (Zhang et al. 2025) ChIP-seq data and the genes designated to cell cycle (Menges et al. 2005), middle layer of regenerative callus (Zhai and Xu 2021), and the GO-term somatic embryogenesis were compared using the Venny online tool (Oliveros 2007–2015). Genes assigned to peaks from the p35S::WOX5-GR ChIP-seq were copied from dataset EV7. Raw data from the pBBM::BBM-YFP ChIP-seq were reanalysed as described (Kerstens et al. 2024), after which genes were assigned to peaks at −3,000 to +500 bp from the transcription start site (Supplementary Data S3), in accordance with the WOX5 ChIP-seq dataset.

### Histology and microscopy

The explants were assessed for Sudan Red staining at 14 d after Dex treatment and 21 d after SIM treatment, respectively. The staining was performed with Sudan Red 7B (Sigma Aldrich) according to previously described protocols (Aichinger et al. 2009; Bouyer et al. 2011). The explants were first dehydrated through an isopropanol series (20%, 40%, and 60%) and incubated for 1 h in 0.5% Sudan Red 7B dissolved in 60% isopropanol solution. After the staining, the plants were rehydrated through the same series in reverse and washed 3 times with water (Aichinger et al. 2009). The samples were observed under a stereoscopic microscope (Nikon SMZ745T).

The ClearSee assays were performed as previously described (Kurihara et al. 2015) with small adjustments. In brief, plant material was immersed in fixation buffer (4% w/v paraformaldehyde in 1× PBS) under vacuum for 1 h and stored at 4 °C for 12 h. After fixation, the explants were washed twice with 1× PBS buffer and then cleared in ClearSee reagent for 1 wk. After staining with 0.1% (v/v) Renaissance SR2200 stain (Renaissance Chemicals Ltd) for 12 h at 4 °C. The stained plants were observed under a Leica inverted confocal microscope (SP8) using a 514 nm laser for YFP and a 405-nm laser for Renaissance.

### Accession numbers

Sequence data from this article can be found in the GenBank/EMBL data libraries under accession numbers that are listed in Supplementary Table S3.

## Supplementary Material

koaf252_Supplementary_Data

## Data Availability

The raw RNA-seq data were deposited at the NCBI SRA database (https://www.ncbi.nlm.nih.gov/sra) under the BioProject accession nr PRJNA1190170.

## Supplementary data

The following materials are available in the online version of this article.

**Supplementary Figure S1.** Induced overexpression of DeDIF and SCN factors.

**Supplementary Figure S2.** Induced overexpression of *WOX5* and *PLT1* separately and transiently in XG-05.

**Supplementary Figure S3.** Time series of transgene-induced regeneration in XG-01 plants.

**Supplementary Figure S4.** Expression profiles of genes related to the cell cycle.

**Supplementary Figure S5.**Expression of cell-type-specific root marker genes during PLT1/WOX5-induced regeneration.

**Supplementary Figure S6.** Expression of callus middle cell layer marker genes during PLT1/WOX5-induced regeneration.

**Supplementary Figure S7.** Induced overexpression of *PLT1/WOX5* activates the somatic embryogenesis pathway.

**Supplementary Figure S8.** Induced overexpression of *PLT1/WOX5* activates LEC1 expression as a prelude to somatic embryogenesis.

**Supplementary Figure S9.** Regeneration of recalcitrant Arabidopsis accessions by induction of SCN gene sets.

**Supplementary Figure S10.** Phylogeny and improved regeneration upon induced LsPLT1/LsWOX5 overexpression in lettuce.

**Supplementary Table S1.** Summary of the selected dedifferentiation (DEDIF) and stem cell niche (SCN) gene sets.

**Supplementary Table S2.** Scoring of regeneration efficiency of different SCN and DEDIF gene combinations.

**Supplementary Table S3.** Genes and identifiers mentioned in this study and those used to create a phylogenetic tree of PLT1, WOX5, and WIND1.

**Supplementary Table S4.** Shoot regeneration and callus formation efficiency of tomato cotyledon explants.

**Supplementary Data S1.** Golden Gate plasmids and primers.

**Supplementary Data S2.** TPM and DEG.

**Supplementary Data S3.** GO_GSEA_GeneLists

## Dive Curated Terms

The following phenotypic, genotypic, and functional terms are of significance to the work described in this paper:

JKD Gramene: AT5G03150
JKD Araport: AT5G03150

LEC1 Gramene: AT1G21970
LEC1 Araport: AT1G21970

LEC2 Gramene: AT1G28300
LEC2 Araport: AT1G28300

RPK1 Gramene: AT1G69270
RPK1 Araport: AT1G69270

WOX2 Gramene: AT5G59340
WOX2 Araport: AT5G59340

WOX7 Gramene: AT5G05770
WOX7 Araport: AT5G05770

OLEO4 Gramene: AT3G27660
OLEO4 Araport: AT3G27660

CUC2 Gramene: At5g53950
CUC2 Araport: At5g53950

FUS3 Gramene: AT3G26790
FUS3 Araport: AT3G26790

PLT1 Gramene: At3G20840
PLT1 Araport: At3G20840

OLEO1 Gramene: AT4G25140
OLEO1 Araport: AT4G25140

ABI3 Gramene: AT3G24650
ABI3 Araport: AT3G24650

ESR1 Gramene: At1g12980
ESR1 Araport: At1g12980

RBR Gramene: AT3G12280
RBR Araport: AT3G12280

ESR2 Gramene: AT1G24590
ESR2 Araport: AT1G24590
