# Supplementary material for: Rational design of induced regeneration via somatic embryogenesis in the absence of exogenous phytohormones
Source: Plant Cell. 2025 Oct 19;37(11):koaf252. doi: 10.1093/plcell/koaf252 (PMC12586336; doi:10.1093/plcell/koaf252)
Supplement: koaf252_Supplementary_Data [file koaf252_supplementary_data.zip › Supplementary Data.pdf]

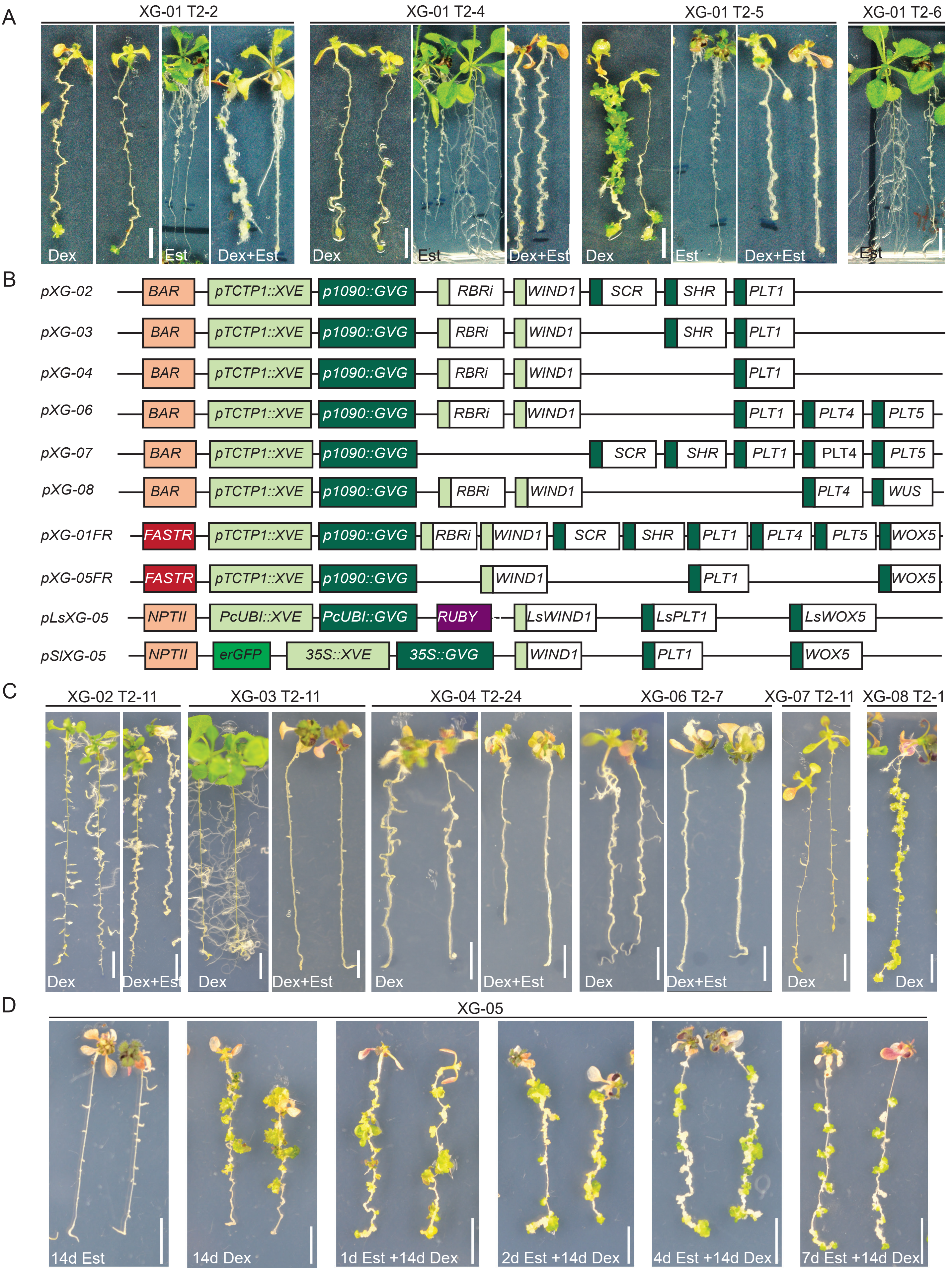

Supplementary Figure S1. Induced overexpression of DeDIF and SCN factors. A) Varying regeneration efficiencies for independent transgenic XG-01 T2 progeny overexpressing *SCN* genes (Dex) or *DeDIF* and *SCN* genes (Est+Dex) at 14dpi. B) Schematic overview of the constructs used in this study. *BAR* and *NPTII* cassettes encodes resistance to phosphinotrycin and kanamycin, respectively. *FASTR*, *RUBY* and *erGFP* cassettes encode visual selection markers. *XVE* and *GVG* represent inducible synthetic transcription factors driving expression of corresponding genes cassettes (green-white bars). *pTCTP1* and *p1090* represent promoter sequences. Ls = Latica sativa, Sl = Solanum lycopersicum, Pc = Petroselinum crispum. C) Formation of callus-like tissue is observed after the induced overexpression of different *DeDIF* and *SCN* gene combinations present in vectors *pXG-02*, *pXG-03*, *pXG-04*, *pXG-06* and *pXG-07* at 14dpi. D) Representative images of XG-05 plants induced with either Est (*WIND1*) or Dex (*PLT1/WOX5*), or sequentially induced with Est for 1d, 2d, 4d or 7d followed by Dex induction for 14 days. Scale bar = 1cm.

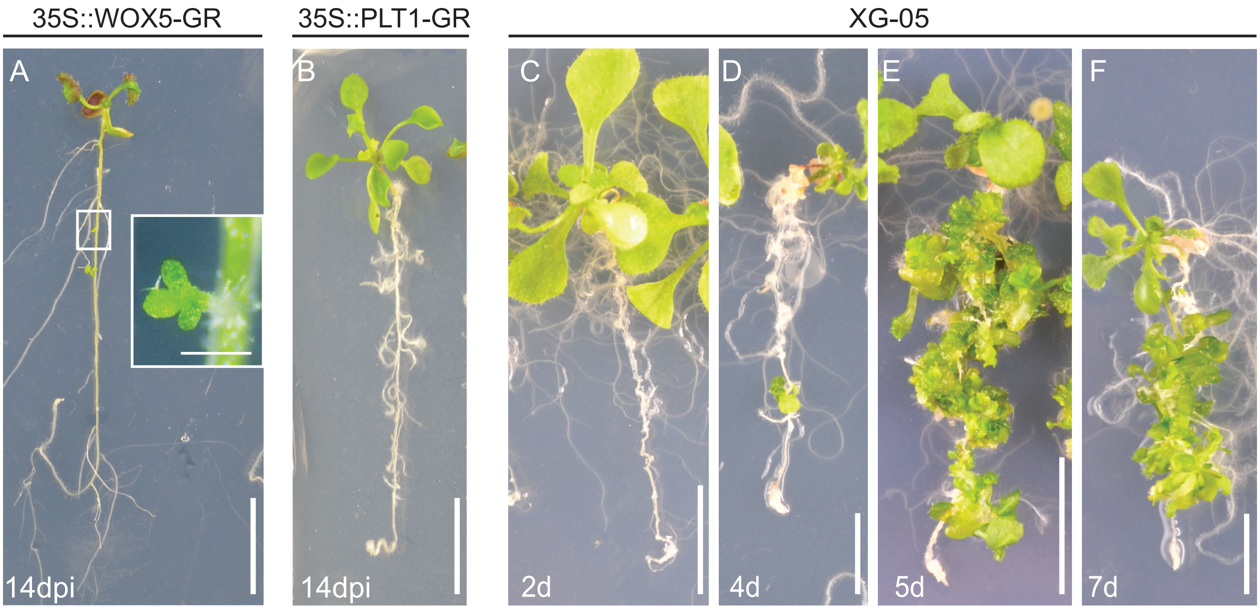

Supplementary Figure S2. Induced overexpression of *WOX5* and *PLT1* separately and transiently in XG-05. A) Induced overexpression of *WOX5* with 10 µM Dex in a 35S::WOX5-GR line led to shoot formation on lateral root tips after 14 dpi. B) Induced overexpression of *PLT1* with 10 µM Dex in a 35S::PLT1-GR line led to root growth stop and callus formation after 14 dpi. C-F) Regeneration response of XG-05 after transient induction of *PLT1* and *WOX5* for 2 (C), 4 (D), 5 (E) and 7 days (F). Scale bar in A-F = 1 cm, inset in A = 50 µm.

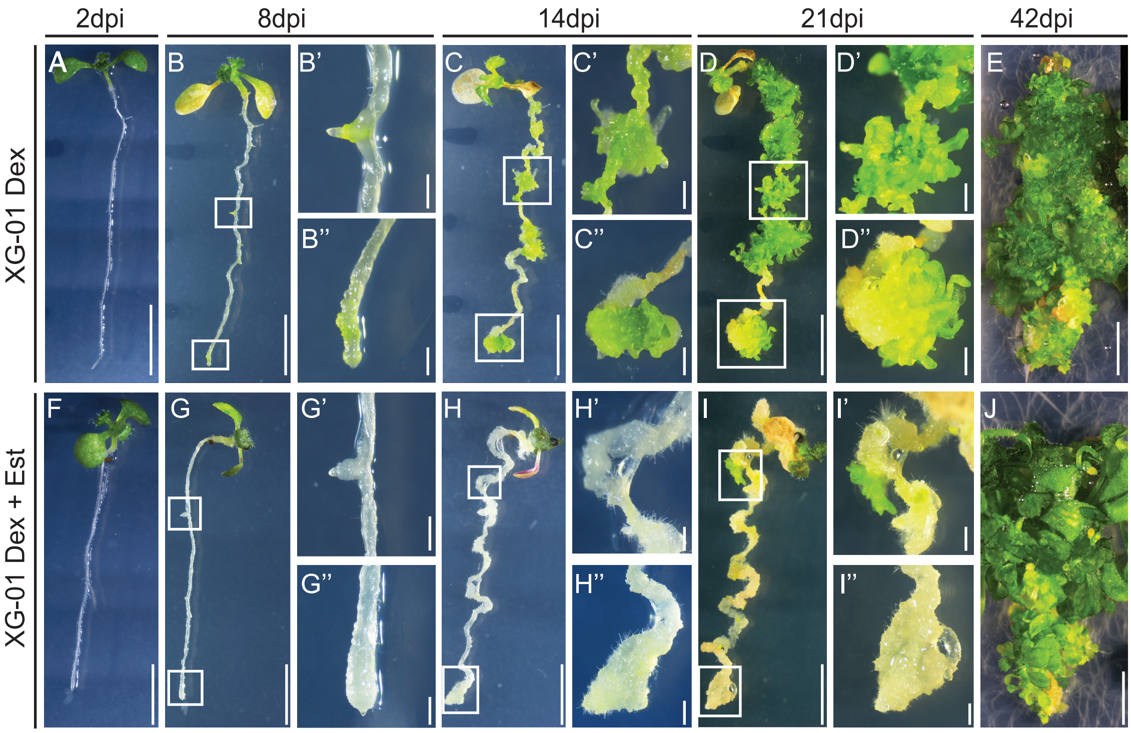

Supplementary Figure S3. Time-series of transgene induced regeneration in XG-01 plants. A-E) Representative images of XG-01 seedlings upon SCN gene set induction with 10 µM Dex, with close ups at 8 (B-B''), 14 (C-C'') and 21 dpi (D-D'') of followed lateral root primordia and root tip. F-J) Representative images of XG-01 seedlings upon DeDIF and SCN gene sets induction with 10 µM Dex and Est, with close ups of followed lateral root primordia and root tip at 8 (G-G''), 14 (H-H'') and 21 dpi (I-I''). The images in panels A, B, C, D, E, F, G, H, I and J is a composite image. Scale bar in A-J = 1 cm, in B'-D', B''-D'', G'-I' and G''-I'' = 1 mm.

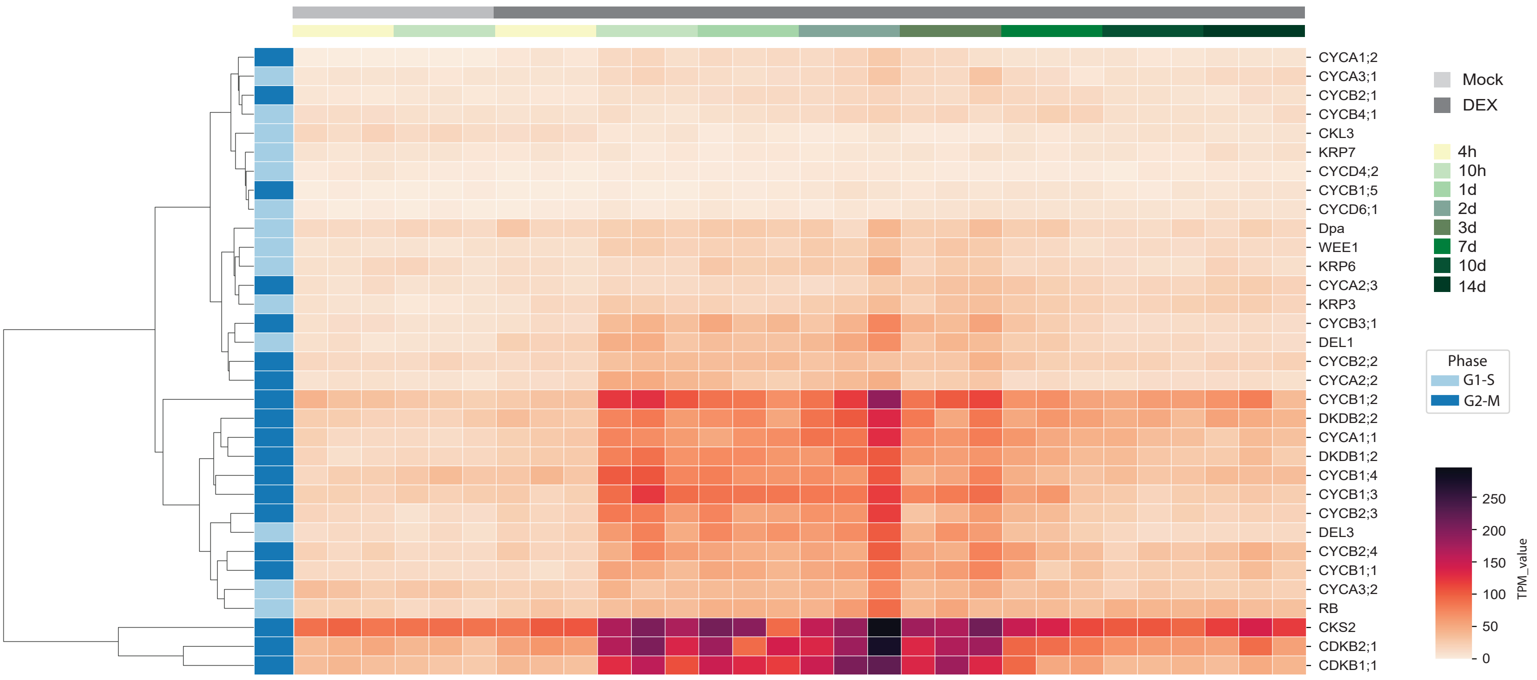

Supplementary Figure S4. Expression profiles of genes related to cell cycle. Expression values in TPM of cell cycle genes involved in G1-S phase (light blue) and G2-M phase (dark blue) of the cell cycle observed in the RNA-seq data of line XG-05 upon induction of *PLT1/WOX5* (Dex) from mock (4h and 10h) to 14 dpi. Each timepoint (yellow to green bars) is represented by 3 biological replicates.

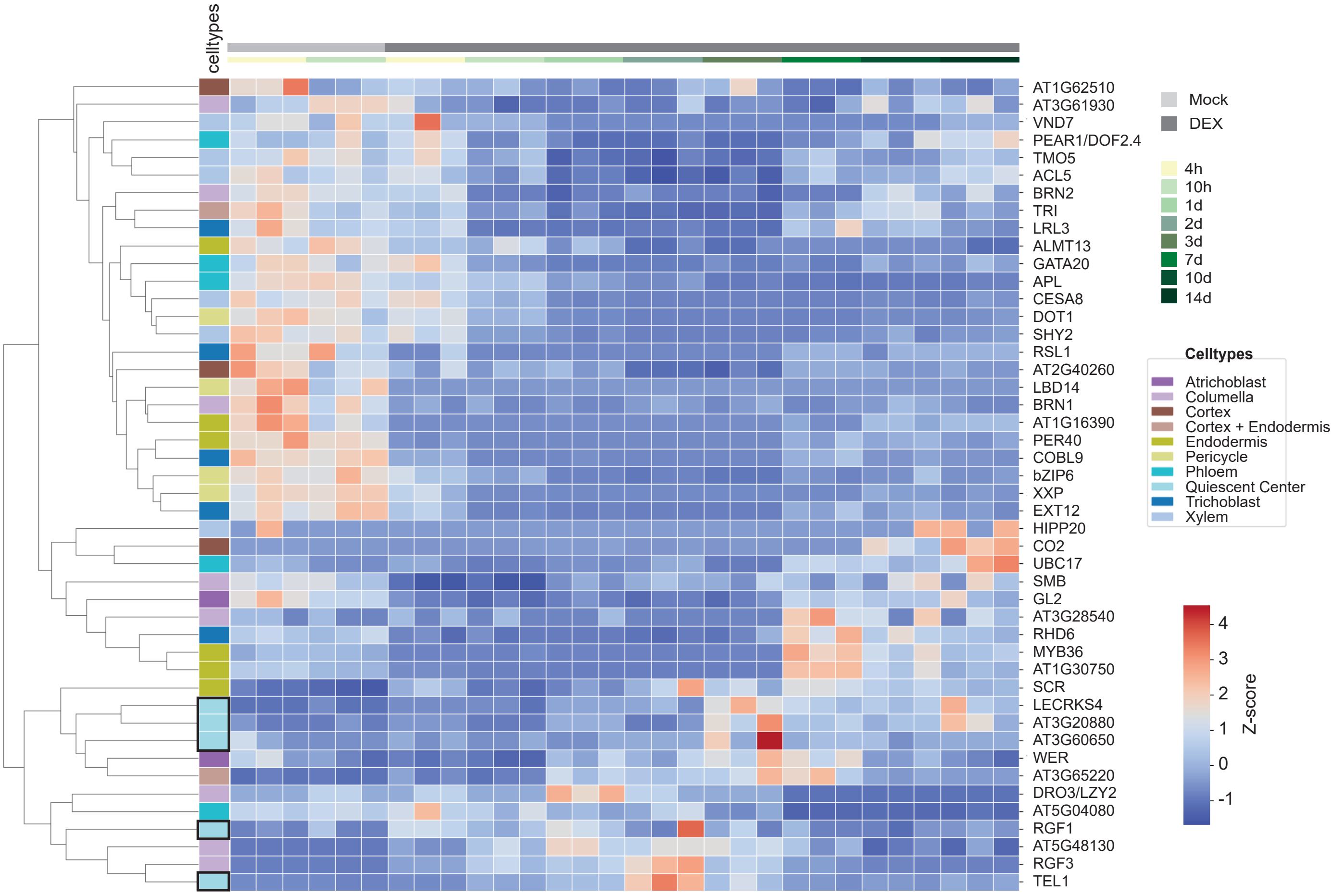

Supplementary Figure S5. Expression of cell type specific root marker genes during PLT1/WOX5 induced regeneration. Expression of root tissue markers (Shahan et al. 2021; Supplementary Data S3) is displayed as Z-score in the RNA-seq data from PLT1/WOX5 induced XG-05 regeneration. Out of 46 marker genes, 38 were downregulated within 1dpi of *PLT1/WOX5* overexpression. Upregulated genes within 3dpi were mainly associated with QC (boxed) and columella identity. Each timepoint (yellow to green bars) is represented by 3 biological replicates.

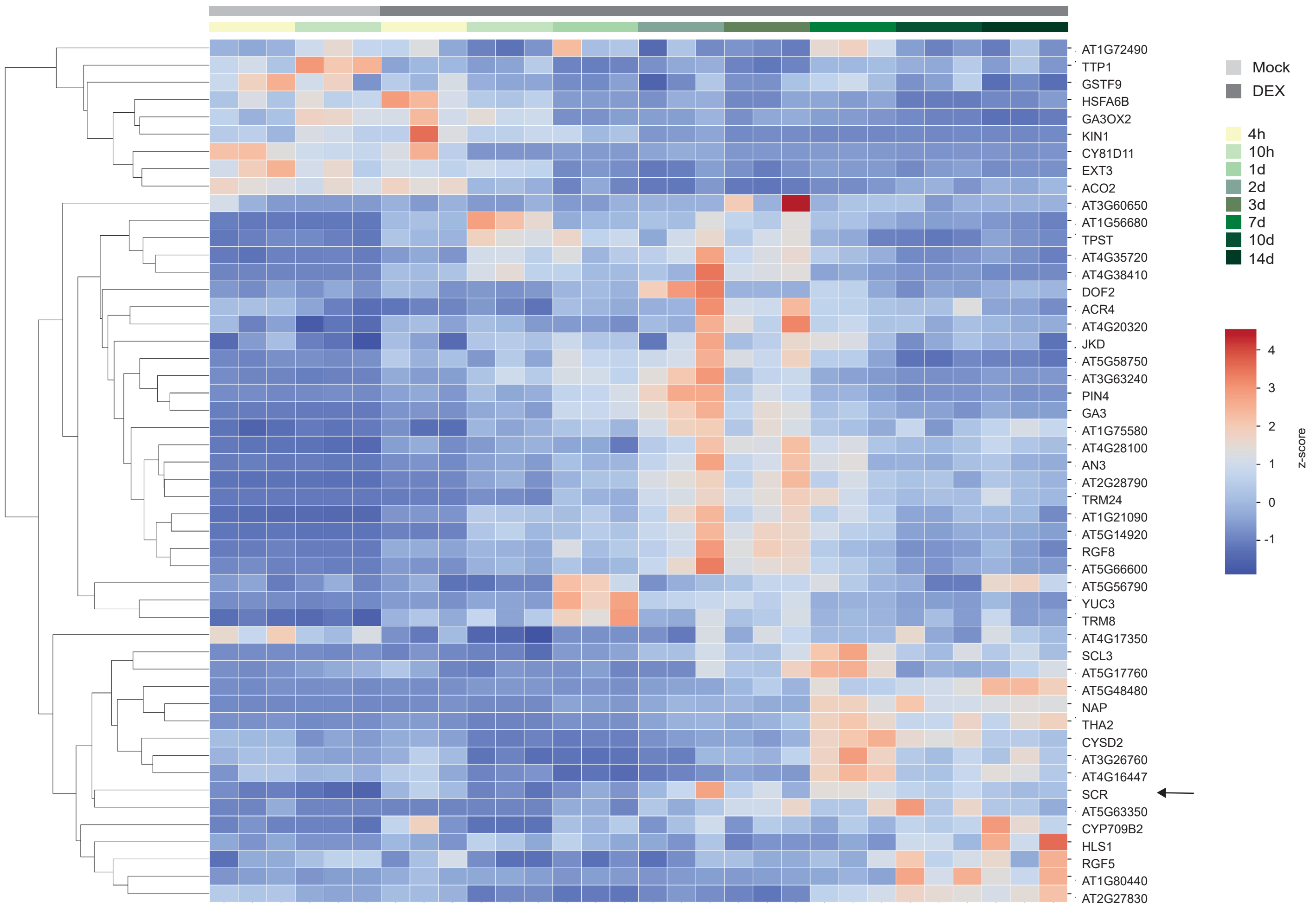

Supplementary Figure 6. Expression of callus middle cell layer marker genes during PLT1/WOX5 induced regeneration. Expression pattern (Z-score) of the top 50 differentially expressed callus middle cell layer genes (Zhai & Xu et al. 2021; Supplementary Data S2) during PLT1/WOX5 induced XG-05 regeneration. Arrow indicates SCR gene expression pattern. Each timepoint (yellow to green bars) is represented by 3 biological replicates.

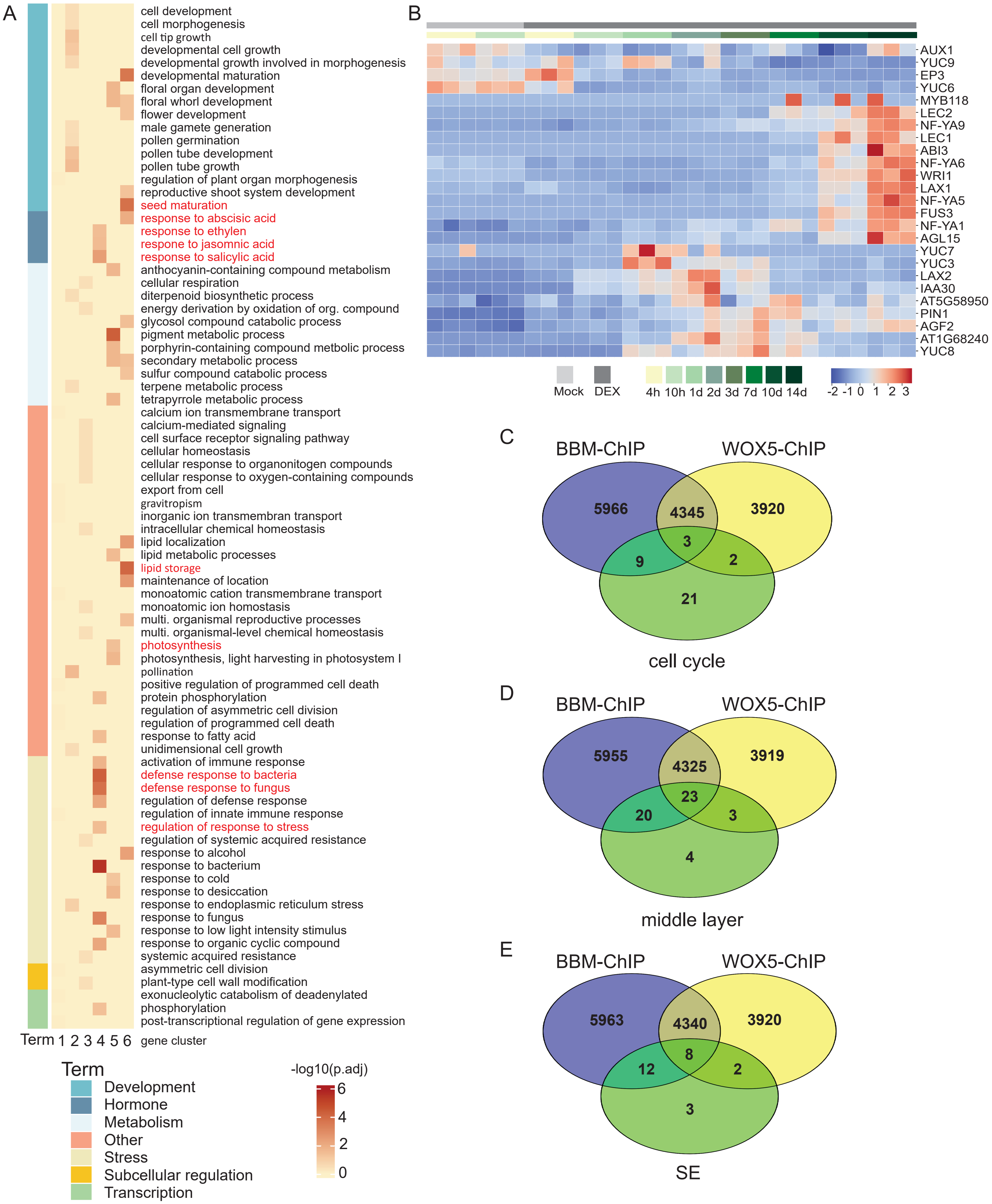

Supplementary Figure S7. Induced overexpression of *PLT1/WOX5* activates the somatic embryogenesis pathway. A) Gene Ontology analysis of gene expression clusters 1-6 (Figure 4E-J). GO terms mentioned in the main text are highlighted in red. P.adj values were calculated by enrichment score statistic as implemented in the fgsea R package (Korotkevich et al., 2019). B) Gene expression profile of genes listed under the GO term somatic embryogenesis. Values are represented as Z-scores. Each timepoint (yellow to green bars) is represented by 3 biological replicates. C-E) Venn diagrams showing the ability of BBM and WOX5 to bind genes related to cell cycle (C), the middle layer of regenerating callus (D) and somatic embryogenesis (E).

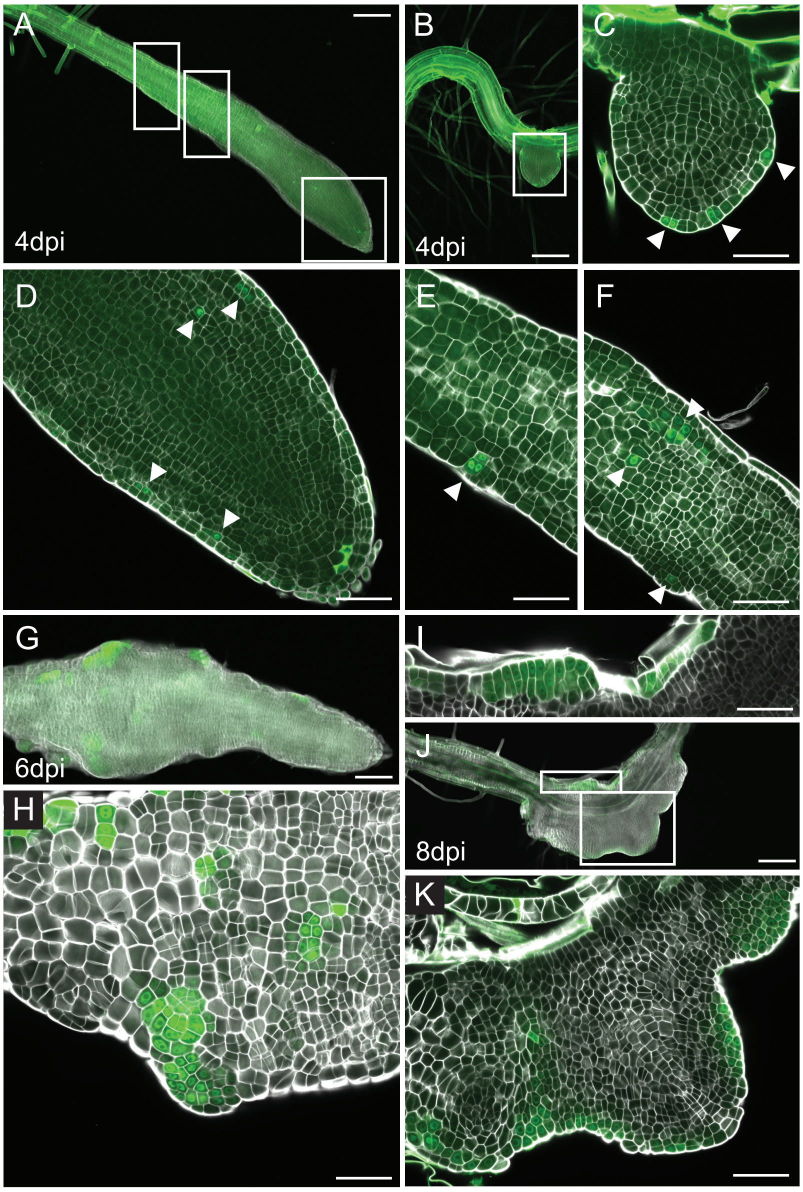

Supplementary Figure S8. Induced overexpression of *PLT1/WOX5* activates *LEC1* expression as a prelude to somatic embryogenesis.
A-K) Parts of regenerating XG-05 seedling roots at 4 dpi (A-F), 6dpi (G,H) and 8dpi (I-K) of *PLT1/WOX5*, showing *pLEC1::LEC1-GFP* expression. Boxes in A correlate to magnifications in D-F, box in B correlates to C and boxes in J correlate to I and K. Arrowheads in C-F indicate sporadic patches of *pLEC1::LEC1-GFP* expression. Scale bar = 100 μm (A, B, G, J) and 50 μm (C-F, H, I, K).

A

		pXG-01FR		*pXG-05FR*	
**Accession**	**Ranking Motte et al. 2014**	**T2 lines**	**Regen. T2 lines**	**T2 lines**	**Regen. T2 lines**
rpk1-5		2	2		
rpki-2		17	5		
rpki-1		12	1		
CIBC-17	88	7	4	22	20
Sq-8	87	6	1	12	11
RRS-7	86	12	6		
UOD-7	84	15	3	8	7
Ga-0	65	16	2	13	10
PNA17	44	12	2		
Col-0	27	12	3		
Fei-0*	13	16	1	6	2
Nok-3*	11	2	0	13	11
Lp2-2*	6	9	2		
Bor-4*	5	19	6	10	8
KZ-9*	4	13	2	8	3
NFA-10	3	16	5		

B

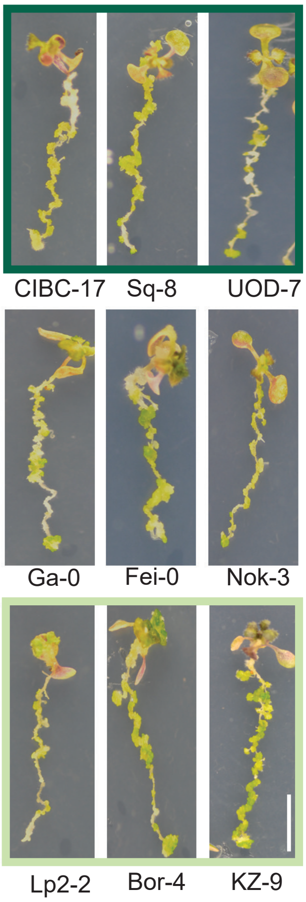

Supplementary Figure S9. Regeneration of recalcitrant Arabidopsis accessions by induction of SCN gene sets. A) Induction of regeneration observed upon overexpression of SCN gene sets in independent T2 transgenic progeny of *pXG-01FR* and *pXG-05FR* transformed selected accessions. Numbers indicate T2 lines produced per accession and portion of these showing regeneration. The Motte ranking (Motte et al., 2014) indicates level of recalcitrance to phytohormone mediated regeneration, with higher numbers being most recalcitrant (dark green) versus lower numbers as highly regenerative accessions (light green). Phytohormone regeneration recalcitrant rpk1 mutants were similarly regeneration upon SCN gene set induction. Accessions marked with an asterisk contain the *RPK1* allele associated with increased regeneration capacity. B) Representative seedlings from experiments in (A) transformed with *pXG-05FR* show regeneration upon PLT1/WOX5 induction scored at 14dpi. Scale bar = 1cm.

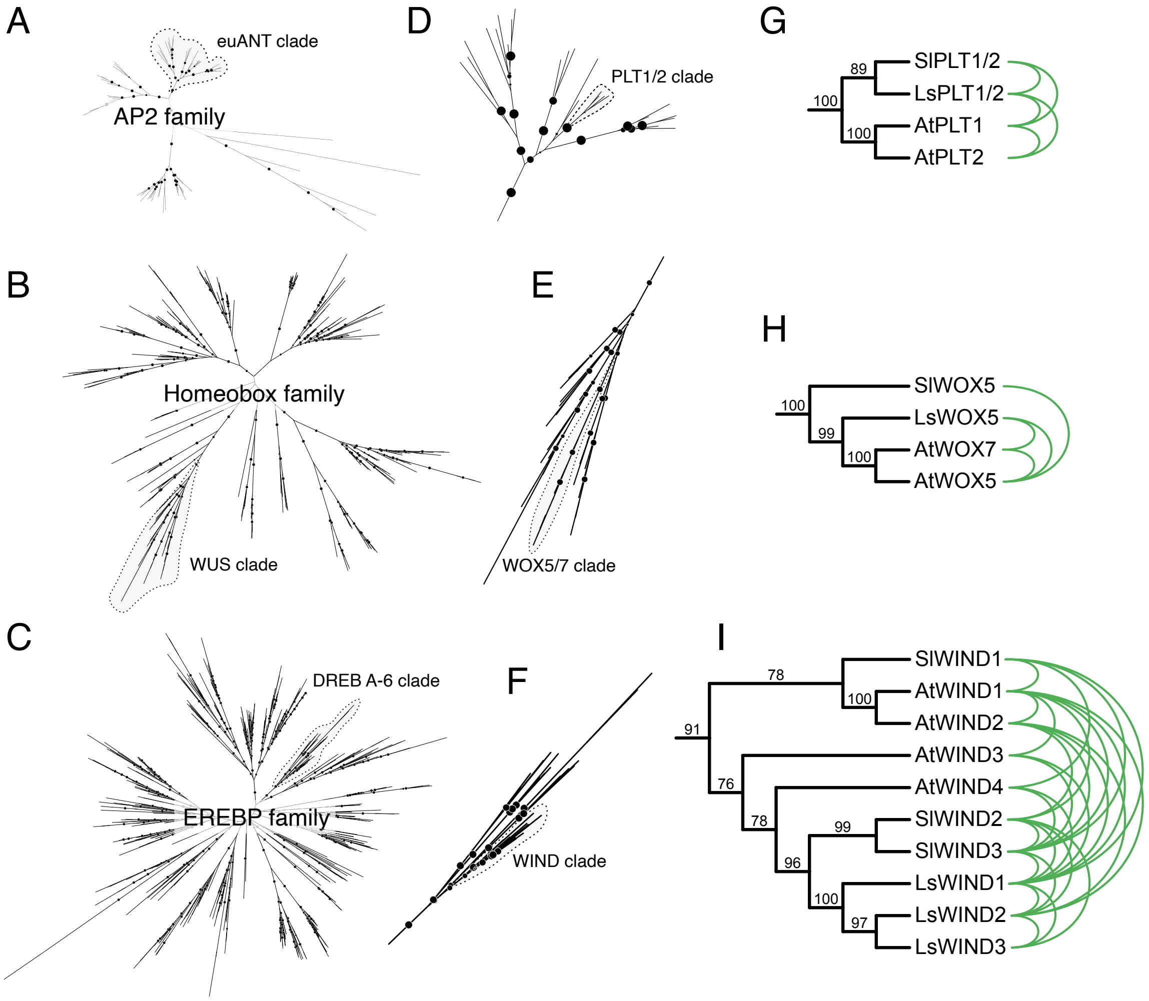

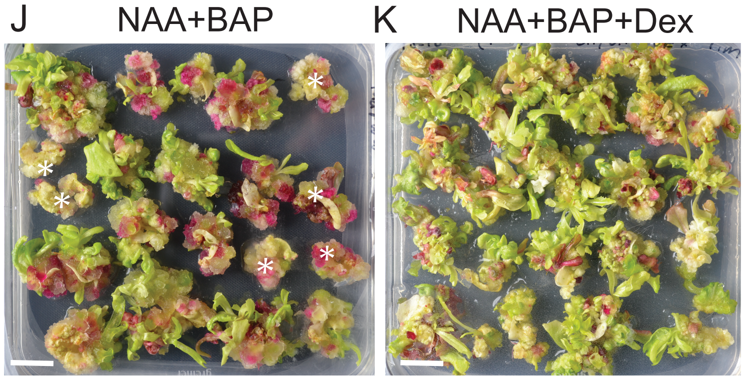

L

Treatment	Exp. 1	Exp. 2
0.1 µM NAA, 0.1 µM BAP	46/66 (77%)	39/100 (39%)
0.1 µM NAA, 0.1 µM BAP, 10 µM Dex	39/40 (98%)**	66/100 (66%)**

Supplementary Figure S10. Phylogeny and improved regeneration upon induced *LsPLT1/LsWOX5* overexpression in lettuce.

A-I) Phylogenetic trees of PLT1, WOX5 and WIND1 based on synteny sequences. A-C) Unrooted phylogenetic tree of AP2 family (A), homeobox family (B) and EREBP family (C) in Arabidopsis, tomato and lettuce. Dots idicate bootstrap values >70. D-F) Magnification of the outlined clades in A-C. G-I) Cladogram of the PLT1/2 clade (G), WOX5/7 clade (H) and WIND clade (I) with indicated bootstrap support, representing outlined regions in D-F. Branch length is ignored. Green curves indicate syntenic connections between genes. J,K) Representative plate of *pLsXG-05*-transformed and regenerating explants at 28 dpi on NAA+BAP (J) and NAA+BAP+Dex (K). Asterisk indicates explant without shoot regeneration (J). L) Quantification of *pLsXG-05*-transformed explants showing regeneratedshoots at 28 dpi with either NAA+BAP or NAA+BAP+Dex in two independent transformation experiments. Statistical difference inshoot-regenerating explants is calculated with a Chi-square test, **=p < 0.001, ***= p<0.0001. Scale bar = 1 cm
