# Supplementary material for: Rational design of induced regeneration via somatic embryogenesis in the absence of exogenous phytohormones
Source: Plant Cell. 2025 Oct 19;37(11):koaf252. doi: 10.1093/plcell/koaf252 (PMC12586336; doi:10.1093/plcell/koaf252)
Supplement: koaf252_Supplementary_Data [file koaf252_supplementary_data.zip › Supplementary_Figure_S1corrected.pdf]

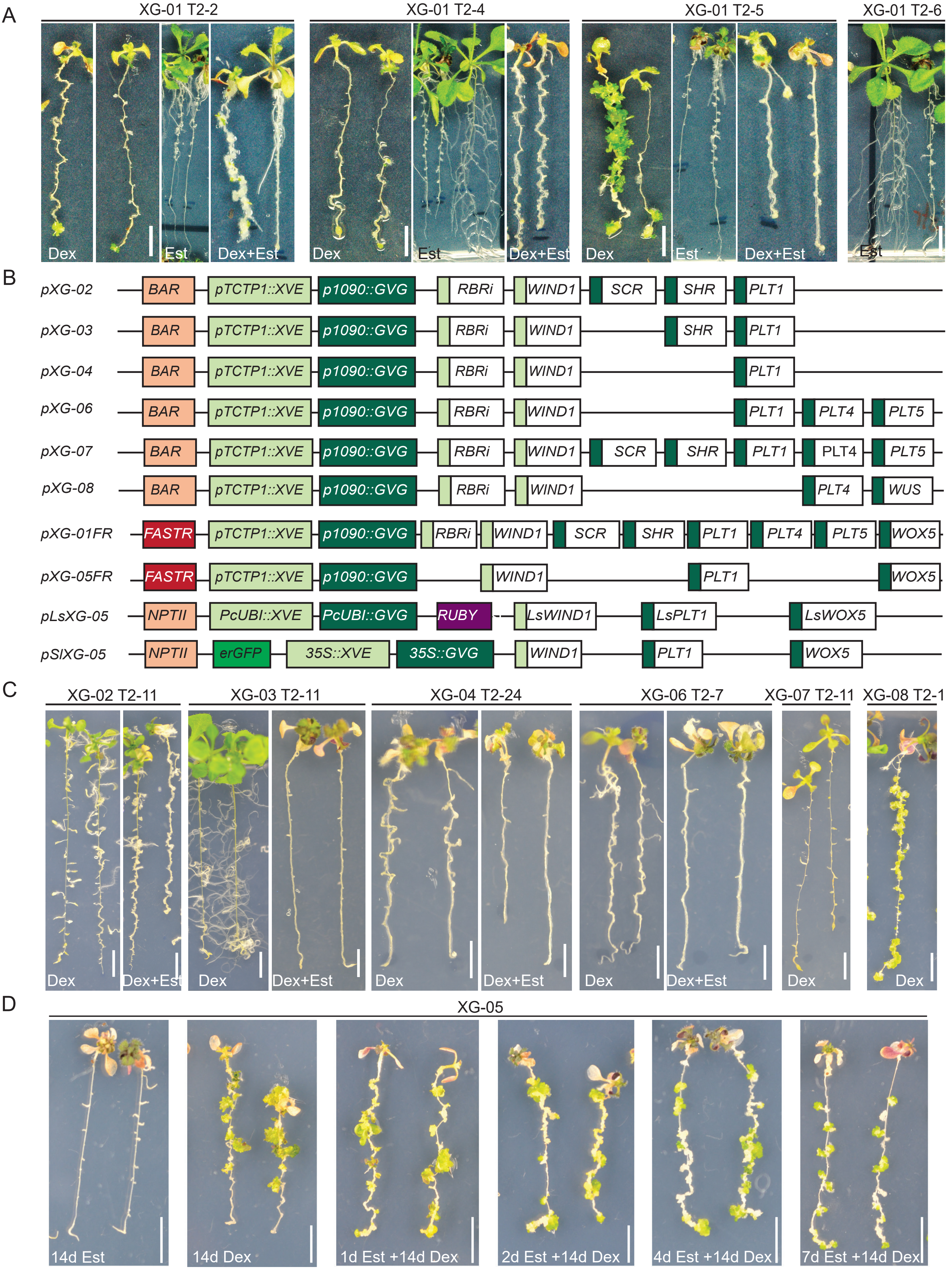


**Supplementary Figure S1. Induced overexpression of DeDIF and SCN factors.** A) Varying regeneration efficiencies for independent transgenic XG-01 T2 progeny overexpressing *SCN* genes (Dex) or *DeDIF* and *SCN* genes (Est+Dex) at 14dpi. B) Schematic overview of the constructs used in this study. *BAR* and *NPTII* cassettes encodes resistance to phosphinotrycin and kanamycin, respectively. *FASTR*, *RUBY* and *erGFP* cassettes encode visual selection markers. *XVE* and *GVG* represent inducible synthetic transcription factors driving expression of corresponding genes cassettes (green-white bars). *pTCTP1* and *p1090* represent promoter sequences. Ls = Latica sativa, Sl = Solanum lycopersicum, Pc = Petroselinum crispum. C) Formation of callus-like tissue is observed after the induced overexpression of different *DeDIF* and *SCN* gene combinations present in vectors *pXG-02*, *pXG-03*, *pXG-04*, *pXG-06* and *pXG-07* at 14dpi. D) Representative images of XG-05 plants induced with either Est (*WIND1*) or Dex (*PLT1/WOX5*), or sequentially induced with Est for 1d, 2d, 4d or 7d followed by Dex induction for 14 days. Scale bar = 1cm.
